# A Europe-wide inventory of citizen-led energy action with data from 29 countries and over 10000 initiatives

**DOI:** 10.1038/s41597-022-01902-5

**Published:** 2023-01-04

**Authors:** August Wierling, Valeria Jana Schwanitz, Jan Pedro Zeiss, Constantin von Beck, Heather Arghandeh Paudler, Ingrid Knutsdotter Koren, Tobias Kraudzun, Timothy Marcroft, Lukas Müller, Zacharias Andreadakis, Chiara Candelise, Simon Dufner, Melake Getabecha, Grete Glaase, Wit Hubert, Veronica Lupi, Sona Majidi, Shirin Mohammadi, Negar Safara Nosar, Yann Robiou du Pont, Philippa Roots, Tadeusz Józef Rudek, Alessandro Sciullo, Gayatri Sehdev, Mehran Ziaabadi, Nahid Zoubin

**Affiliations:** 1 Western Norway University of Applied Sciences, Department of Environmental Sciences, Sogndal, 6856 Norway; 2 The Schumacher Institute, The Create Centre, Bristol, BS1 6XN United Kingdom; 3 GREEN Research Centre, Bocconi University, Via Röntgen 1, 20136 Milan, Italy; 4 Institute of Sociology, Jagiellonian University in Kraków, Ul, Grodzka 52, 31-044 Kraków, Poland; 5 Department of Culture, Politics, and Society, University of Turin, Via Verdi 8, 10124 Turin, Italy

**Keywords:** Energy and society, Energy supply and demand

## Abstract

Numerous case studies show that citizens engage in various ways in renewable and low carbon energy projects, thereby contributing to the sustainable energy transition. To date, however, a systematic and cross-country database on citizen-led initiatives and projects is lacking. By performing a major compilation and reviewing copious data sources from websites to official registries, we provide a Europe-wide inventory with over 10,000 initiatives and 16,000 production units in 29 countries, focusing on the past 20 years. Our data allow cross-country statistical analysis, supporting the elicitation of empirical insights capable of extending beyond the perspective of single case studies. Our data also align with ongoing efforts to implement two EU Directives that aim at strengthening the active role of citizens in the energy transition. While the focus of our data collection is on Europe, the data and methodology can contribute to the global analysis of citizen-led energy action.

## Background & Summary

The Paris Agreement signed in 2015 states that greenhouse gas emissions resulting from human activities must be reduced as soon as possible in order to limit global warming well below 2°C compared to pre-industrial levels by 2100^1^. This will require a rapid low carbon transition in almost all sectors of human activity, and in particular reaching net zero emissions for the production, distribution, and consumption of energy services^2^.

In many countries, ordinary citizens are coming together through collective initiatives to play an active role in this transition. Particularly in Europe, citizen-led energy projects have grown to produce, distribute, and consume energy from renewable sources while being governed democratically, with benefits accruing locally^3^. While many of these initiatives are small in scope, they are of sufficient importance to policymakers as they actively involve people in the transformation^4–6^. Under the name of energy communities, citizen-led energy action has been specifically addressed in two separate EU directives (Directives EU-2018/2001^7^ and EU-2019/944^8^). Despite this, data collection on the topic has not been undertaken systematically until now. Notable exceptions include Harnmeijer *et. al*.^9^, Harnmeijer *et. al*.^10^, Haggett *et al*.^11^, Hewitt *et al*.^12^, Kahla *et al*.^13^, Hoicka and MacArthur 2018^14^, Wierling *et al*.^15^, Gorrono-Albizu *et al*.^16^, and Berka *et al*.^17^. Haggett *et al*.^11^ is an early inventory on citizen-led action in Scotland. While Kahla *et al*.^13^ collects long-term data on German citizen-led energy cooperatives and associations (‘Bürgerenergiegesellschaften und Energiegenossenschaften’), Hewitt *et al*.^12^ carry out a mapping exercise, compiling over 400 community energy initiatives in 8 European countries. Wierling *et al*.^15^ is the precursor to the inventory presented here. The majority of the research done on the phenomenon of citizen-led energy action, however, takes the form of case studies, surveys, or compendia (c.f. Holstenkamp 2018^18^). Such studies provide meaningful insights drawn from smaller sets of examples, but fail to give an understanding of the aggregate contributions of these initiatives. Additionally, Non-Governmental Organizations (NGOs) in some European countries that support, structure, and track national citizen energy movement have provided analyses of the impact of these actions (c.f. RESCOOP.eu - the European Federation of Citizen Energy Cooperatives). To this point, the analyses do not provide a comprehensive review of citizen-led climate action in Europe based on a dataset driven approach. While the data produced in this manner has been invaluable to the present effort, it is neither exhaustive nor sufficiently detailed to support in-depth analysis of the sector. Therefore, the research community has been calling for dataset driven approaches (Harnmeijer *et. al*.^9^, Wierling *et al*.^15^, Hewitt *et al*.^12^).

**Fig. 1:**
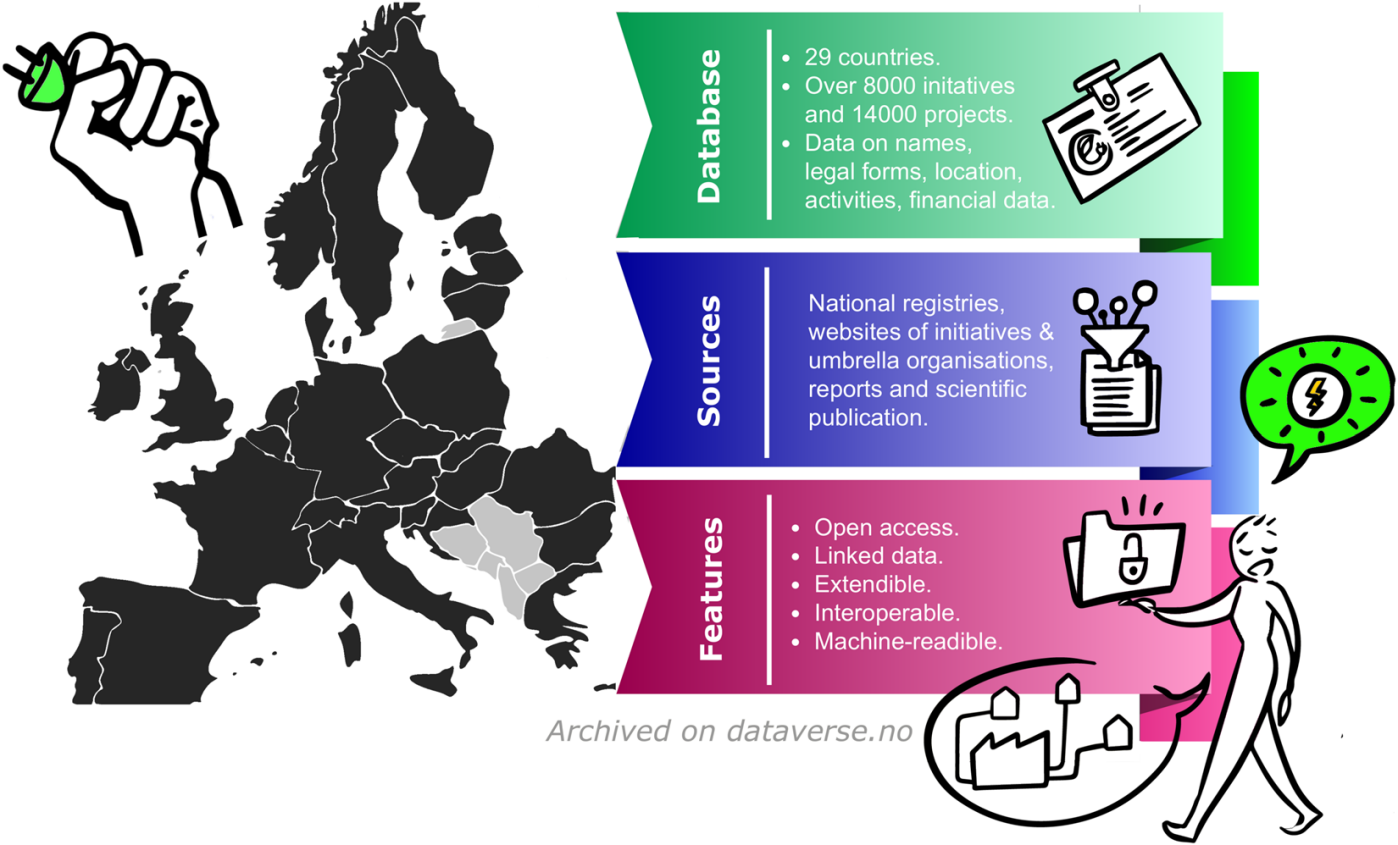
Overview on the EU-wide inventory of citizens collectively engaging in the energy transition.

**Fig. 2:**
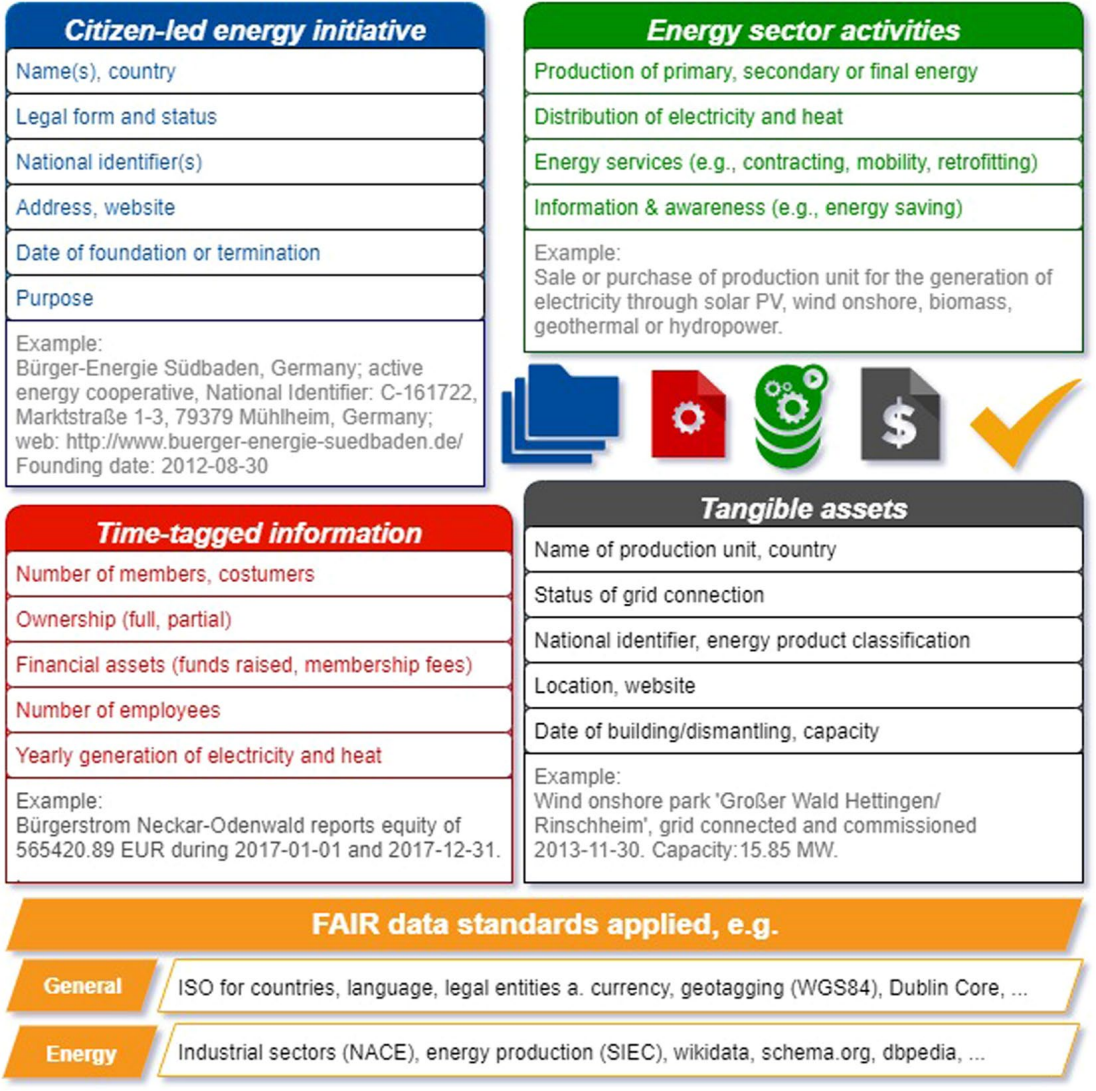
Schema of the inventory on citizen-led energy initiatives in 329 European countries. The figure gives examples about the type of information gathered. For the taxonomy and definitions of terms refer to the Supplementary Material.

The data presented here is the first to capture the nature and scope of collective citizen-led action in the energy transition for each country in Europe (Fig. 1). The data consists of a broad range of variables to a high degree of granularity, covering both organizations and the individual projects that they manage, e.g., units under own operation to produce renewable electricity or the operation of charging stations for electric mobility. This dataset draws on official registries, umbrella organization databases, news reports, websites of initiatives, and both individual organization legal documents (bylaws, meeting minutes, etc.), social media, and websites. Collection was performed over a period of four years by an international team of trained researchers and assistants and validated both manually and through automated processes. Figure 2 provides a schematic overview of the design of the inventory, which we detail in the remainder of this paper. An additional publication will complement the documentation of the database by detailing the steps undertaken to implement the FAIR guiding principles^19^ for machine-actionable reuse of the data. These 15 principles suggest organizational steps to increase the findability (F), accessibility (A), interoperability (I), and reusability (R) of data.

Being the first of its kind, the Europe-wide inventory of citizen-led energy initiatives can be valuable to all key actors concerned with citizen engagement in the energy transition, from policymakers to academics to advocacy organizations to the citizen-practitioners themselves. The dataset allows generalizable conclusions to be drawn both within and between countries, permitting comparisons across both time and borders. Of particular value to public authorities and legislators, these analyses give insights into the impacts of citizen initiatives on the energy transition and of policy decisions on those same initiatives. This data will be particularly relevant over the next few years within public policy, as the member states of the EU implement the directives involving Energy Communities into local law and track the impacts of these policy changes on the emergence and success of citizen-led initiatives in their jurisdictions.

A systematic analysis of the enabling and disabling factors for these initiatives is now achievable with this dataset for the first time by comparing between countries and within a given country across time. This data can be used to support the construction of likely trajectories that citizen engagement will take and to make recommendations for how to alter or improve those trajectories. Citizen-led projects themselves and their network organizations can also benefit from this dataset, strengthening their ability to advocate for their position in the energy transition and helping them to learn from the experiences of initiatives in other countries. The aggregated contribution of energy communities to the energy transition at the national and European level can be documented with the help of the data. According to these estimates, at least 2 million people invested their time, creativity, and money into the installation of about 10 GW of renewable capacity. While the focus of our data collection is on Europe, the data can also support empirical analysis of citizen-led energy action around the globe.

## Methods

### Defining criteria for citizen-led renewable energy initiatives

The literature provides a wide array of terms and definitions for citizen-led renewable energy initiatives, ranging from ‘community renewable energy projects (CREs)^20^’, ‘local low-carbon energy initiatives (LLCEIs)^21^, to ‘grassroots energy initiatives (GIs)’^22^. Recently, the European Commission has published two related definitions, being Citizen Energy Communities (CEC)^7^ and Renewable Energy Communities (REC)^8^. As a result of the various definitions, this paper adopted a broad conceptualization, aiming to be over-inclusive rather than under-inclusive. The inventory allows filtering based on, for example, the legal form of the initiative, to provide future users with the possibility to select sub-samples of the dataset that fit their own criteria. Relevance of initiatives for the inventory is decided based on three aspects,

1. the initiative being led by citizens,
2. the initiative striving for social or environmental benefit beyond pure economic interest, and
3. the initiative engaging in activities related to the energy transition.

Activities considered are not only production and distribution of energy, but also include actions such as information and awareness raising. Note that the inventory reports collective citizen initiatives and not initiatives of individual citizens.

For the purpose of defining the aspects of ‘citizen-led’ and additional social or environmental benefits, we adopted the concept of process and outcome dimensions by Walker and Devine-Wright (2008)^20^, where the process dimension refers to the degree of participation of the initiative’s members in its decision-making and the outcome dimension refers to the degree to which the initiative focuses on providing community benefits. Thereby, *community* is understood as communities of place and/or interest^23^. The highest degree of participation in the initiative’s decision making process is given for cases following the ‘one member - one vote’ principle’ (OMOV principle). Here, in comparison to companies such as shareholdings, every member has one vote, independent of the share owned by the respective member. For the majority of countries in Europe, the ‘one member - one vote’ principle applies to the legal form of cooperatives^24,25^. Consequently, typical actors included in the inventory are cooperatives under the individual national law. Other legal forms were included for specific countries if evidence suggested a high degree of participation in decision-making processes. Legal forms were identified and categorized using the Entity Legal Form Codes provided by the Global Legal Entity Identifier Foundation (GLEIF)^26^. Table 1 summarizes the included legal forms for each country.

**Table 1:** Relevant legal forms, GLEIF legal entity codes, and information on prevailing of the One-Member-One-Vote (OMOV) Principle in a country.

Country	Legal Forms – with ✓ (✓) or (✓) or x in-front indicating adherence to the OMOV-principle	GLEIFIdentifier
AUT	✓ Registrierte Genossenschaft (m.b.H.) (registered cooperative), ✓Verein (association)	8XDW, DX6Z
BEL	✓ Société coopérative (cooperative), ✓ Société coopérative à responsabilité limitée (limited liability cooperative), [✓] Société coopérative à responsabilité limitée à finalité sociale (social benefit limited liability cooperative), ✓ Société coopérative européenne (European cooperative), (✓) Association sans but lucratif (non-profit association), (✓) Association internationale sans but lucratif (international non-profit association)	28FE, 8E2A, 2QSA, YBHM, W3WH, V03J
BGR	[✓] Drushestwo s ogranitschena otgowornost (limited liability company). Other potentially relevant forms: Kooperazija (cooperative), Sdrushenie w obschtschestwena polsa (social benefit association)	VJ3G, CTCH, 3SHX
HRV	✓ Zadruge/Zadruga (cooperative)	G5RH
CYP	No legal forms for citizen-led energy actions. Cooperative law also established in Cyprus	N/A
CZE	✓ Družstvo (cooperative). Another possible relevant legal form: Zemědělské družstvo (collective farm)	9RVC, Z3BF
DNK	Interessentskab (partnership), Andelsselskab med begrænset ansvar (limited liability organisation)	7WRN, PZ6Y
EST	(✓) Korteriühistu (apartment association), ✓ Mittetulundusühing (non-profit association), ✓Tulundusühistu (commercial association)	8ZQE, PRTB, VSEV
FIN	✓ /[✓] Osuuskunta (cooperative)	EE90
FRA	(✓) SAS société par actions simplifiée (Simplified joint-stock company), [✓] SARL nationale (limited liability corporation), ✓ SCOP SARL coopérative ouvrià¨re de production (worker cooperative), Association fonciére urbaine (urban real-estate association), Association déclarée (association), ✓ Association déclarée reconnue d’utilité publique (registered public benefit association), ✓ Association non déclarée (undeclared association), [✓] SICA SARL d’intérêt collectif agricole (collective interest limited liability agricultural company), Société coopérative agricole (agricultural cooperative), [✓] Indivision entre personnes physiques (partnership between physical persons), Autre SA coopérative à conseil d’administration (other joint-stock cooperative), Autre SARL coopérative (other limited liability cooperative)	6CHY, JR7T, V1Z5, 9O0S, BEWI, XH8C, 491H, H3ZD, 211B, Q634, 8II5, 4DZU
DEU	✓ Eingetragene Genossenschaft (registered cooperative company), [✓] Eingetragener Verein (registered association)	US8E, QZ3L
GRC	✓ but limited citizen leadership E*v*εργεiα*kή* Koi*vó*τητα (Energy Community - specific sub-form of a cooperative)	J3VJ
HUN	Yet limited activities in citizen energy, but current relevant legal form: Egyéb szövetkezet (Other cooperatives)	DPY1
IRE	(✓) Friendly Society, no legal form: Sustainable Energy Communities	54SK, n/a
ITA	Associazione (association), Società Cooperativa (cooperative), Società Semplice (simplified company), Società Consortile A Responsabilità Limitata (limited liability consortium), Società Cooperativa a Responsabilità Limitata (Limited liability cooperative), Consorzio (consortium), Società A Responsabilità Limitata (limited liability company), Società Per Azioni (joint-stock company), Cooperativa Sociale (social cooperative)	1TON, QRZJ, 2XXH, BL52, CTNS, HN75, OV32, P418, PHMS
LVA	No GLEIF registered legal forms, but citizens engage in: ✓ Biedriba (society), ✓ Kooperatīvā sabiedrība (Cooperative society)	8888 8888
LTU	Only one registered GLEIF code: ✓ Kooperatinės bendrovės (Cooperatives). People also engage in Housing associations	LUGM 8888
LUX	[✓] Société civile (civil company), ✓ Société coopérative (cooperative society), (✓) Association sans but lucratif (non-profit association), (✓) Société coopérative organisée comme une société anonyme	SQ1A, V5OS, 2JEI, STBC
MLT	✓ Cooperative society	F5X7
NLD	✓ Coöperatie (cooperative), (✓) Vereniging (association, society), [✓] Stichting (foundation, trust)	NFFH, 33MN, V44D
NOR	✓ Samvirkeforetak (cooperative), ✓ Europeisk samvirkeforetak (european cooperative)	K5P8, O7LB
POL	(✓) but limited citizen leadership SpóÅ‚dzielnie (cooperative), informal concept of “Energy Clusters”	8TOF, n/a
PRT	✓ Cooperativa (cooperative), associação	1HGD, ALPT
ROU	✓ Societate Cooperativă Europeană (European cooperative society)	UWEE
SVK	✓ Družstvo (cooperative)	I7AS
SVN	✓ Zadruga z omejeno odgovornostjo (cooperative limited liability)	RAX7
ESP	✓ Sociedad cooperativa (cooperative)	1QU8
SWE	✓ Ekonomisk förening (economic association), [✓] Samfällighetsförening (joint-ownership association), ✓ Bostadsförening (housing association before 1930), ✓ Bostadsrättsförening (tenant owner’s association after 1930), [✓] Ideell förening (non-profit organization), [✓] Enkla bolag (regulated partnership between two partners)	C61P, n/a, WZDB, SSOM, 1TN0, n/a
CHE	✓ Genossenschaft, Société coopérative, Società cooperativa (cooperative)	QSI2
GBR	✓ Community benefit society, company, community interest company, LLP	IYXU, H0PO, 17R0, Z0EY

Information on OMOV sourced from EPRS^25^. Abbreviations: (✓) - optional, but common choice, [✓] - or alternatively also proportional, multiple, plural votes, ((✓)) for cooperatives of natural persons. Note: 8888 means pending status.

The following areas of activity have been classified as relevant for engagement in the energy transition (see also Table 2):

- Production and distribution of energy: Comprising the operation, installation, and/or financing of any kind of renewable energy generation facility (solar-PV, wind, hydro-power, geothermal, bio-energy), distribution of electricity or heat, energy trade, collective purchasing of energy and energy-related products, and the production and trade of energy-related products (such as energy crops);
- Provision of energy services: Including low carbon self-consumption, contracting of light, car sharing and operation of EV charging stations, bike sharing, retrofitting of buildings, and energy efficiency and energy saving measures (including the operation/installation/financing of co-generation units using fossil fuels); and
- Information & awareness: Such as energy-related education and awareness raising and energy consulting services.

**Table 2:** List of applied classification standards and controlled vocabularies.

Standard	Name and use
SIEC^35^	Standard International Energy Product Classification
GLEIF^26^	Global Legal Entity Identifier Foundation
ISO 3166^36^	Alpha-2 and alpha-3 country codes
ISO 639-1^37^	Codes for the representation of names of languages — Part 1: Alpha-2 code
ISO 4217^38^	International currency codes
NACE^32^	Statistical classification of economic activities of the European Union
schema.org^39^	Shared vocabularies for structured data on the internet.
dbpedia^40^	Structured information used to encode city names or tangible assets
wikidata^41^	Structured information used to encode semantic information, e.g., wikidata Q194356 of a “wind farm”
OEO^42^	Open Energy Ontology

Additional taxonomic details are provided in the Supplementary Material. We follow ISO-25964 (The international standard for thesauri and interoperability with other vocabularies).

### Data search and extraction process

Data collection extended over a period of four years, from 2018 to 2021, sourcing from four overarching categories of data sources. These include 1) pre-existing registries and databases, 2) individual websites of the initiatives, 3) regular (financial) accounts and reports of the initiatives, and 4) direct interviews with members of the initiatives or experts in the field of citizen-led renewable energy initiatives. Curated data sources include official registries operated by state agencies such as official business registers and energy market data registers (e.g., the German Marktstammdatenregister^27^, Swedish Vindbrukskollen^28^, Danish Datavirk^29^), commercial/non-profit/scientific databases such as commercial business registers and pre-existing databases on community energy operated by NGOs and other non-governmental actors (e.g Dutch Lokale Energie Monitor^30^, database of the European federation of citizen energy cooperatives (REScoop)^31^), and lastly data from previous studies related to citizen-led renewable energy initiatives. Detailed information on these sources per country are provided in the Supplementary Material. Official registries, as well as commercial/non-profit/scientific databases, tend to be dynamically updated, while previous studies tend to provide static data collected within a given time period. In summary, priority was given to official registries operated by state agencies, followed by other curated databases and static data from previous studies. Thereafter, individual initiatives’ websites, yearly accounts, peer-reviewed publications, and interviews with experts were used to fill in missing data and to validate the data already collected.

An initial meta-study was conducted for each of the investigated countries to identify relevant legal frameworks (e.g., legal forms for initiatives), previous literature on the topic of citizen-led, collective energy action, and potential data sources. If available, any existing data specifically on citizen-led renewable energy initiatives was thereafter added to the inventory. In such cases, all data obtained through this process was cross-referenced with official registries and/or individual initiatives’ websites and yearly accounts and potentially updated. Following this step, a systematic search in business registries was conducted, expanding the previous data. Business registries where generally searched by filtering all entries for the relevant legal forms as well as keywords related to the energy transition. Ideally, business registries offer an integrated option to filter for both legal forms and area of activity (by following the NACE^32^ or similar classification systems). For countries where business registers do not provide this option, a web search by keywords was conducted instead. Details for each country are provided in the Supplementary Material. Some countries maintain additional registries on actors engaging in the energy sector, such as the German Marktstammdatenregister that contains information on all installed energy generation facilities. If such registries were available, these were searched for all entries fitting the relevant legal forms of the actors. Following the process of identifying relevant initiatives, data was collected from the aforementioned sources.

## Data Records

The data have initially been compiled in spreadsheets/csv-files, but subsequently transferred into records, applying the research description framework (RDF). The turtle format^33^ was used as RDF serialization. Four top-level classes (or digital units) were used to organize the data (see Fig. 2), where each of them includes attribution information following the Dubling Core Standard. Top-level data classes are the following:

1. Administrative data on citizen-led energy initiatives: Name (legal, alternatives), status (active/inactive), isLegalOrganization (yes/no), National Identifier(s), country code, postal address, contact, website, year of foundation, year of termination, legal form (as text and as code), industrial sector classification (national and international sector classification, e.g., for the production of electricity), One-member-one-vote (connected to legal forms, refer to Table 1), purpose statement (text, English and/or national language), makesOffer (specification of areas of activity in the energy sector);
2. Tangible assets: Name of production unit, status (active/inactive), IsGridConnected (yes/no), geotagged location, street address, country, national production unit identifier(s), technology and energy product identifier (e.g., photovoltaic), year of commissioning, year of decommissioning, nameplate capacity, estimated yearly generation, equipment specification;
3. Singular activities undertaken by an initiative: For example, the purchase and sale of tangible assets (dates, values/prices), organisation of events; and
4. Time-tagged information, with yearly resolution: Number of members, number of customers, number of employees, financial assets (total assets, fixed assets, tangible assets, current assets, total equity and liabilities, equity), percentage ownership of assets, yearly generation of electricity or heat per production unit.

The inventory is publicly accessible at dataverse.no and adheres to the FAIR data guiding principles^19^. Technical details about the FAIRification process will be described in a complimentary publication. This implies that the inventory, as well as the instances of the four classes, are described through rich metadata and linked with persistent identifiers. Controlled vocabularies and ontologies have been used to define the metadata and assign classification standards. Table 2 lists these standards. Further information about additional uses of country standards and classification is available in the Supplementary Material.

The inventory on citizen-led energy initiatives is publicly open from the general purpose repository dataverse.no^34^

## Technical Validation

As a general validation measure, the four-eyes principle was strictly implemented. This implies that the person in charge of collecting the data was not the same for validating the data. Where possible, data have been cross-checked with other publications and aggregated information. Also, statistical tests were performed, such as verification of the possible range of data. Detailed information about validation undertaken country-by-country is available in the Supplementary Material.

Due to different legislation and reporting requirements in each of the countries, the coverage of data varies considerably. We can report the tendency that more detailed information is available for larger initiatives. Furthermore, legal forms are not yet available or under implementation in some countries (see also Table 1), which is why we are only able to report very basic information. In the Supplementary Material we provide assessments on the data coverage within each of the countries, using available literature or other information as benchmarks. Overall, above 70 percent of the initiatives were officially registered and over 70 percent have a website established. Information about members and production units is available for 40 percent and 50 percent respectively. Countries with the best coverage include Belgium, Denmark, Germany, and the Netherlands, whereas much less information is available from the Czech Republic, Finland, Croatia, and Switzerland.

Our dataset falls short in covering a number of initiatives and their activities for the following reasons. First, it is likely that we were not able to identify all initiatives that predate 1980 but still exist today (e.g., historic electrical cooperatives). Second, we are not able to cover all dissolved initiatives that lack a trackable web presence or have been deleted from official registers. In general, the coverage of initiatives and their activities in a given country is best if it requires official registration. For example, registration is typically required for initiatives engaging in the production of electricity, whereas neither initiatives nor activities of housing associations need formal or standardized recording. Therefore, our data likely underestimate the role of housing associations, which play an important role in Eastern European countries. Third, we exclusively rely on voluntary information available from websites or other media for collecting data on soft activities such as information and awareness raising, education, and consultancy services. Therefore, we are likely underestimating the contribution of collective action in this regard, overemphasizing initiatives engaging in the production of energy services.

## Usage Notes

The inventory on citizen-led energy initiatives in European countries is available for reuse under the Creative Common licence CC-BY 4.0. Licensees may copy, distribute, display, perform, and make derivative works and remixes based on it only if they give credit (attribution) to the authors by citing this manuscript.

Although we put utmost attention to ensure high quality data (see the Section above), the inventory should be reused with caution. The concept of energy communities or citizen-led action in the energy transition is constantly evolving and cross-country monitoring standards do not yet exist. Moreover, obligations for registering and/or updating existing initiatives and projects differs across countries, which is why the data are incomplete. Thus, the numbers may serve as conservative estimates, able to provide country- and European-level snapshots.

## Supplementary information

Supplementary material to the manuscript

## Data Availability

No custom code was used to generate or process the data described in the manuscript. Rather, a combination of open software tools was applied. However, the supplementary material published at the repository^34^ contains customized SPARQL commands to query the inventory.
