# Supplementary material for: A Europe-wide inventory of citizen-led energy action with data from 29 countries and over 10000 initiatives
Source: Sci Data. 2023 Jan 4;10:9. doi: 10.1038/s41597-022-01902-5 (PMC9813342; doi:10.1038/s41597-022-01902-5)
Supplement: Supplementary file 1 — Supplementary material to the manuscript [file 41597_2022_1902_MOESM1_ESM.pdf]

17.06.2022

Supplementary material to the manuscript

"A Europe-wide inventory of citizen-led energy action, data from 29 countries and over 10000 initiatives"

Authors: *August Wierling, Valeria Jana Schwanitz, Jan Pedro Zeiss, Constantin von Beck, Heather Arghandeh Paudler, Ingrid Knutsdotter Koren, Tobias Kraudzun, Timothy Marcroft, Lukas Müller, Zacharias Andreadakis, Chiara Candelise, Simon Dufner, Melake Getabecha, Grete Glaase, Wit Hubert, Veronica Lupi, Sona Majidi, Shirin Mohammadi, Negar Safara Nosar, Yann Robio du Pont, Philippa Roots, Tadeusz Józef Rudek, Alessandro Sciullo, Gayatri Sehdev, Mehran Ziyabadi, Nahid Zoubin.*

Abstract: This appendix provides details for the data collection for each country. We provide details on 1) the terminologies used in the database, 2) Details for each country about data collection and validation (incl. list of search terms, keywords, filter criteria; procedures for ensuring data validation and completeness; contributors; references for main data sources and other literature used to complete and/or validate country data), and 3) Guidelines on how to access the database. For main data sources, we specify the type of source (O = other, OR = Official registers by government authorities, DB = other databases not run by government authorities, such as commercial registers, R = reports, P = Publications). For example, BEL_1_O stands for the first main datasource for Belgium which is classified as "other". The provenance of data is additionally documented in the database file itself, following FAIR guiding principles (see also Wierling and Schwanitz 2022).

1 Database - definition of terms 5
Terms for energy sector activities 6
Production 6
Solar 6
Photovoltaics 6
Concentrated solar 6
Thermal 6
Wind 6
Wind onshore 6
Wind offshore 6
Hydropower 7
Geothermal 7
Geothermal deep 7
Geothermal shallow 7
Cogeneration 7
Biofuels 7
Distribution 7
Grid operation 7
Electricity 8
Heat 8
Gas 8
Water 8
ICT 8

Trade 8
Electricity 8
Biogas 8
Tenant solutions 8
Self-consumption 9
Storage 9
Energy services 9
Retro-fitting 9
Supporting self-consumption 9
Contracting 9
Mobility 9
Operation and maintenance of EV-charging stations 9
Car sharing 10
Car pooling 10
Other 10
Consulting 10
Information and awareness 10
Training 10
Finances and funding 10
Planning 10
Information 10
Other 11
Agriculture 11
Housing 11
Finance and banking 11
Other 11
Terms for energy services technologies 11

Austria (AUT) 11
Methodological approach 11
Validation & completeness 12
Contributors 12
References 12

Belgium (BEL) 12
Methodological approach 12
Validation & completeness 13
Contributors 13
References 13

Bulgaria (BGR) 14
Contributors 14
References 14

Croatia (HRV) **15**
Methodological approach 15
Validation & completeness 15
Contributors 15
References 15

Cyprus (CYP) **16**
Methodological approach 16
Validation & completeness 16
Contributors 16
References 16

Czech Republic (CZE) **17**
Methodological approach 17
Validation & completeness 17
Contributors 17
References 17

Denmark (DNK) **17**
Methodological approach 17
Validation & completeness 18
Contributors 18
References 18

Estonia (EST) **19**
Methodological approach 19
Validation & completeness 19
Contributors 19
References 20

Finland (FIN) **20**
Methodological approach 20
Validation & completeness 20
Contributors 20
References 21

France (FRA) **21**
Methodological approach 22
Validation & completeness 22
Contributors 22
References 22

Germany (DEU) **23**
Methodological approach 23
Validation & completeness 24

Contributors 24

References 24

Great Britain (GBR) 25

Methodological approach 26

Validation & completeness 26

Contributors 26

References 26

Greece (GRC) 26

Methodological approach 26

Validation & completeness 27

Contributors 27

References 27

Hungary (HUN) 28

Methodological approach 28

Validation & completeness 28

Contributors 28

References 28

Ireland (IRL) 28

Methodological approach 28

Validation & completeness 29

Contributors 29

References 29

Italy (ITA) 30

Methodological approach 30

Validation & completeness 30

Contributors 30

References 30

Latvia (LVA) 31

Methodological approach 31

Contributors 32

References 32

Lithuania (LTU) 32

Methodological approach 33

Validation & completeness 33

Contributors 33

References 33

Luxembourg (LUX) 33

Methodological approach 34

Validation & completeness 34
Contributors 34
References 34

Malta (MLT) 35
Methodological approach 35
Validation & completeness 35
Contributors 35
References 35

Netherlands (NLD) 36
Methodological approach 36
Validation & completeness 36
Contributors 36
References 36

Norway (NOR) 37
Methodological approach 37
Contributors 38
References 38

Poland (POL) 38
Methodological approach 38
Validation & completeness 38
Contributors 38
References 38

Portugal (PRT) 39
Methodological approach 39
Validation & completeness 39
Contributors 39
References 39

Romania (ROU) 40
Methodological approach 40
Validation & completeness. 40
Contributors 40
References 40

Slovakia (SVK) 41
Methodological approach 41
Validation & completeness 41
Contributors 41
References 41

Slovenia (SVN) 41

Methodological approach 42
Validation & completeness 42
Contributors 42
References 42

Spain (ESP) **42**
Methodological approach 43
Contributors 43
References 43

Sweden (SWE) **43**
Methodological approach 43
Validation & completeness 44
Contributors 44
References 44

Switzerland (CHE) **45**
Methodological approach 45
Validation & completeness 45
Contributors 45
References 46

3 Guidelines to access the database **46**
TTL triples and SPARQL query language 46
Useful SPARQL commands for database queries, country-level 49
Useful SPARQL commands for database queries, initiative-level 54
Other useful SPARQL commands for database queries 55
Extended PREFIX and variable lists for applied standards 57

1 Database - definition of terms

The ontology related to the ENBP inventory is organized as a turtle file, and is used to describe data on citizens engaging in the energy transition. It supports machine-actionable access to definitions and connected standards with persistent identifiers. The ontology is available at dataverse.no where the inventory is also stored:

Wierling, August; Schwanitz, Valeria Jana; Zeiss, Jan Pedro; von Beck, Constantin; Arghandeh Paudler, Heather; Knutsdotter Koren, Ingrid; Kraudzun, Tobias; Marcroft, Timothy; Müller, Lukas; Andreadakis, Zacharias; Candelise, Chiara; Dufner, Simon; Getabecha, Melake; Glaase, Grete; Hubert, Wit; Lupi, Veronica; Majidi, Sona; Mohammadi, Shirin; Safara Nosar, Negar; Robio du Pont, Yann; Roots, Philippa; Rudek, Tadeusz Józef; Sciullo, Alessandro; Sehdev, Gayatri; Ziaabadi, Mehran; Zoubin, Nahid, 2022, "ENBP Inventory "Energy by people" - First Europe-wide inventory on energy communities", https://doi.org/10.18710/2CPQHQ.

The following section provides the taxonomic structure of the database, which has energy sector activities as the topterm. The structure includes the definition of all terms and their hierarchy in the taxonomy, following ISO-25964. We use:

BT - broader term (e.g., as solar is to PV).
NT - narrower term (e.g., as PV is to solar)
DEF - text defining the term itself
DA - date of definition

In addition, we use vocabularies defined by statistical offices (e.g., economic sector activity codes - NACE, classifications for energy production defined in SIEC). Finally, we implement machine-actionability for the inventory which requires definitions that are machine-accessible and readable. The details for implementing the FAIR data principles are documented in Wierling and Schwanitz 2022.

Terms for energy sector activities

<u>Production</u>

DA 2022-01-10
DEF Production of primary, secondary, or final energy
BT Energy sector activity
NT Solar
NT Geothermal
NT Wind
NT Biomass

Solar

DA 2022-01-10
DEF Electricity generation based on the conversion of light
BT Production
NT Photovoltaics
NT Concentrated solar
NT Thermal

Photovoltaics

DA 2022-01-10
DEF Electricity generation based on the conversion of light using semiconductor materials
BT Solar

Concentrated solar

DA 2022-01-10
DEF Electricity generation from concentrated sunlight using mirrors or lenses
BT Solar

Thermal

DA 2022-01-10
DEF Heat generation from light using solar thermal collectors
BT Solar

Wind

DA 2022-01-10
DEF Electricity generation based on the conversion of kinetic energy of the wind
BT Production
NT Wind onshore
NT Wind offshore

Wind onshore

DA 2022-01-10
DEF Electricity generation based on wind technology located at land
BT Wind

Wind offshore

DA 2022-01-10
DEF Electricity generation based on wind technology located at sea
BT Wind

Hydropower

DA 2022-01-10
DEF Electricity generation based on the conversion of kinetic energy of flowing water
BT Production

Geothermal

DA 2022-01-10
DEF Electricity generation based on the conversion of thermal energy of the earth
BT Production
NT Geothermal shallow
NT Geothermal deep

Geothermal deep

DA 2022-01-10
DEF Electricity or heat generation based on thermal energy in the earth crust
BT Geothermal

Geothermal shallow

DA 2022-01-10
DEF Heat generation using ground source heat pumps
BT Geothermal

Cogeneration

DA 2022-01-10
DEF Simultaneous generation of electricity and heat
BT Production

Biofuels

DA 2022-01-10
DEF Production of fuels, electricity, or heat coming from biomass
BT Production

<u>Distribution</u>

DA 2022-01-10
DEF Activities including the operation of grids (electricity, water, heat, ICT), trade in the energy sector (electricity, biogas, tenant solutions), storage solutions, and energy self-consumption
BT Energy sector activity
NT Grid operation
NT Trade
NT Self-consumption

NT Storage

Grid operation

DA 2022-01-10
DEF Operation of grids to supply electricity, heat or water
BT Distribution
NT Electricity
NT Heat
NT Gas
NT Water
NT ICT

Electricity

DA 2022-01-10
DEF Operation of local electricity grids
BT Grid operation

Heat

DA 2022-01-10
DEF Operation of local district heating networks
BT Grid operation

Gas

DA 2022-01-10
DEF Operation of local gas grids
BT Grid operation

Water

DA 2022-01-10
DEF Operation of local water (incl. sewage) networks
BT Grid operation

ICT

DA 2022-01-10
DEF Operation of broadband internet services
BT Grid operation

Trade

DA 2022-01-10
DEF Trade with energy-related products and services
BT Distribution
NT Electricity
NT Biogas
NT Tenant solutions

Electricity

DA 2022-01-10
DEF Trade of electricity at spot markets and to customers
BT Grid operation
NT Spot markets
NT Customers

Biogas

DA	2022-01-10
DEF	Trade of biogas
BT	Grid operation

Tenant solutions

DA	2022-01-10
DEF	Offering tenant solutions (e.g., tenant's sub-metered electricity supply)
BT	Grid operation

Self-consumption

DA	2022-01-10
DEF	Operation of an electricity production units for self-consumption purposes
BT	Distribution

Storage

DA	2022-01-10
DEF	Operation of an energy storage solution
BT	Distribution

Energy services

DA	2022-01-10
DEF	Offering energy services, e.g. contracting, retro-fitting of buildings, mobility.
BT	Energy sector activity
NT	Retro-fitting
NT	Contracting
NT	Mobility
NT	Consulting

Retro-fitting

DA	2022-01-10
DEF	Offering measures to improve the energy efficiency of buildings
BT	Energy services

Supporting self-consumption

DA	2022-01-10
DEF	Offering consultation services in support of self-consumption
BT	Energy services

Contracting

DA	2022-01-10
DEF	Offering contracting services to manage improved energy-efficiency solutions (e.g., contracting to switch to LED-street light, renewable-based heat)
BT	Energy services

Mobility

DA	2022-01-10
DEF	Offering mobility services
BT	Energy services

NT Operation and maintenance of EV-charging stations
NT Car sharing
NT Car pooling
NT Other (e.g., ticket trading, ride hailing, e-bike rental and others not included yet)

Operation and maintenance of EV-charging stations
DA 2022-01-10
DEF Operation and maintenance of EV charging stations
BT Mobility

Car sharing
DA 2022-01-10
DEF Offering and managing the sharing of cars through rental services
BT Mobility

Car pooling
DA 2022-01-10
DEF Operating carpools to share rides
BT Mobility

Other
DA 2022-01-10
DEF Other activities in the mobility sector not yet covered (ticket trading, ride hailing, e-bike rental)
BT Mobility

Consulting
DA 2022-01-10
DEF Offering consulting services in the energy sector
BT Energy services

Information and awareness
DA 2022-01-10
DEF Informing and raising awareness about the low-carbon energy transition in the local community; other activities.
BT Energy sector activity
NT Training
NT Finances and funding
NT Planning
NT Information

Training
DA 2022-01-10
DEF Offering training to increase knowledge of the low-carbon energy transition
BT Information and awareness

Finances and funding
DA 2022-01-10
DEF Organizing events for the local community to build knowledge about possibilities to fund and/or finance low-carbon energy transition projects
BT Information and awareness

Planning

DA	2022-01-10
DEF	Organizing events for the local community to strengthen planning capacities
BT	Information and awareness

Information

DA	2022-01-10
DEF	Organizing general information events for the local community to strengthen the active participation of citizens in the energy transition
BT	Information and awareness

Other

DA	2022-01-10
DEF	Other activities
BT	Activities in other sectors
NT	Agriculture
NT	Housing
NT	Finance and banking
NT	Other

Agriculture

DA	2022-01-10
DEF	Agricultural activities, incl. growing energy crops
BT	Other

Housing

DA	2022-01-10
DEF	Housing, incl. real estate
BT	Other

Finance and banking

DA	2022-01-10
DEF	Finance and banking activities
BT	Other

Other

DA	2022-01-10
DEF	Other, e.g., tourism
BT	Other

Terms for energy services technologies

Allowed terms for energy services technologies include production technologies (solar - photovoltaics, concentrated solar, thermal; wind - onshore, offshore; hydropower; geothermal - shallow, deep; cogeneration; biofuels), energy storage and mobility (EV-charging). For the sake of simplicity, we refrain here from repeating the controlled vocabulary following ISO-25964.

Austria (AUT)

Methodological approach

Cooperatives were identified based on three main sources. These are, first, the official business register, partly accessible on firmenabc.at (AUT_1_DB), second, the AGRAR PLUS report (AUT_2_P), and, third, a map of Austrian power plants (AUT_3_O). Information on production units was partly available in publications of RE associations summarizing production, and extended by other search results on the internet, individual websites, and newspaper articles (AUT_4_O). Furthermore, reference brochures of RE development companies and manufacturers were consulted.

Validation & completeness

Data accuracy: Cross-referencing between news articles and website information, if available. **Data collection errors:** Application of the four eyes principle and cross checks between different data sources. **Validation of aggregates/Representativeness of sample:** Our data are in agreement with trends from Seiwald (AUT_5_R) and (AUT_6_O). **Further remarks:** The Austrian data sample mostly consists of district heating initiatives, also active in the agricultural supply sector. Most of the initiatives have few members and do not maintain their own websites. Information is thus often conveyed through local newspapers or umbrella organization reports.

Contributors

Constantin von Beck, Tobias Kraudzun, August Wierling, Simon Dufner, Jan Pedro Zeiss

References

AUT_1_DB, Web service mirroring the Austrian business registry, https://www.firmenabc.at

AUT_2_P, AGRAR PLUS report 150. Heizwerk in Niederöstereich, https://agrarplus.at/folder-broschueren.html?file=files/agrarplus-inhalte/folder_broschueren/folder_150_heizwerk.pdf&cid=127

AUT_3_O, Map of Austrian power plants, https://openinframap.org/stats/area/Austria/plants

AUT_4_O, Initiatives websites, newspaper articles, social media, etc.

AUT_5_R, The (up) scaling of renewable energy technologies: experiences from the Austrian biomass district heating niche, https://doi.org/10.2478/mgr-2014-0011

AUT_6_O, AGRAR PLUS report 200, Heizwerk in Niederöstereich, https://agrarplus.at/folder-broschueren.html?file=files/agrarplus-inhalte/folder_broschueren/folder200_heizwerk.pdf&cid=126

Belgium (BEL)

Methodological approach

Walloon Region: The initial identification of cooperatives was based on a list of REScoop members (BEL_2_O) and through a search for energy-related terms in a list of all registered cooperatives provided by the Ministry of Economy (BEL_3_OR). Further information was collected from the Belgian official business register (BEL_4_OR) through a search for entries with the relevant legal form (see Tab. 1 in the manuscript) and NACE industrial sector codes. Basic administrative information was collected from BEL_4_OR, with individual initiatives listings providing links to Belgian National Bank records (BEL_5_OR), and also to legal filings such as statutes in the National Gazette (BEL_6_OR). Finally, we conducted snowball sampling through mentions of further initiatives on websites and social media of already identified initiatives. Cross-referencing of basic administrative data and collection of additional data on energy production units was based on the Initiative's Websites (BEL_1_O). Search terms used for BEL_3_OR: Energie/Energy; Citoyen/Citoyenne; Eole/Eolienne; Vent/Wind; Moulin; Hydro; Chaleur/Chauffage/Chaud; Electricité/Electricity/Elec; Watt; Courant; Ener; ENR: Renouvelable: Durable: Transition; Biogaz; Métha**.**

Flemish region: The initial identification of cooperatives was based on a list of REScoop members (BEL_2_O). In addition to the snow balling method and data sources as used for identifying data for the Walloon region, we additionally used the official registry Kruispuntbank van Ondernemingen (KBO) - BEL_8_OR.

Validation & completeness

Data accuracy: If available, cross-checking of administrative and financial data from business register (BEL_4_OR) and national bank (BEL_5_OR) with information on initiatives' websites. Production data could not be cross-referenced as only available on initiatives' websites. **Data collection errors:** Four eyes principle, automated check for duplicates and wrong data formats during generation of data implementation files. **Validation of aggregates/representativeness of sample:** We count a total of 109 initiatives and 586 production units. Comparison with previous publications: The REScoop Flanders website (BEL_7_O) reports as of 11/02/2022 24 member cooperatives, while the REScoop Wallonia website (BEL_2_O) lists 20. Bauwens, Gotchev, & Holstenkamp (2016) report a total of 34 relevant organizations for the country and Holemans & Van de Velde report 27 renewable energy cooperatives in Belgium in 2017. Our findings show at least 51 such groups with a founding date prior to 2016. These discrepancies can be explained by the more inclusive nature of our criteria, as it includes organizations which act in other dimensions of the energy transition (renovation, education, energy efficiency) rather than only focusing on production as these other sources do. In addition, our dataset lists each legal structure separately, even when they are a part of a tightly-related group of legal entities while the studies and sources listed above generally aggregate such instances into single listings. These differences suggest that our dataset includes all of those initiatives previously studied, including some that have not been, and contributes to a higher degree of confidence in the completeness of our data.

Contributors

Timothy Marcroft, August Wierling, Constantin von Beck

References

BEL_1_O, Initiatives' websites, (social) media, n/a

BEL_2_O, RESCoop Wallonie Website, https://www.rescoop-wallonie.be

BEL_3_OR, Economy ministry list of all registered cooperatives in Belgium, https://economie.fgov.be/sites/default/files/Files/Entreprises/Liste-des-cooperatives-agreees.pdf

BEL_4_OR, Economy ministry public search of business registry, https://economie.fgov.be/fr/themes/entreprises/banque-carrefour-des/services-pour-tous/banque-carrefour-des-0

BEL_5_OR, Belgian Central Bank Balance Sheet Office, https://cri.nbb.be/bc9/web/

BEL_6_OR, Belgian National Gazette, http://www.ejustice.just.fgov.be/cgi/summary.pl

BEL_7_O, RESCoop Flanders Website, https://www.rescoopv.be/

BEL_8_OR, Kruispuntbank van Ondernemingen (KBO) https://kbopub.economie.fgov.be/kbopub/toonondernemingps.html?ondernemingsnummer=715938489

Additional references

Bauwens, T. , Gotchev, B. & Holstenkamp, L. What drives the development of community energy in Europe? The case of wind power cooperatives. *Energy research & social science.* 13, 136-147; https://doi.org/10.1016/j.erss.2015.12.016 (2016).

Holemans, D., Van de Velde, K. *Citizens energy: Making energy democracy happen*. Green European foundation & OIKOS report. https://gef.eu/wp-content/uploads/2019/01/GEF_Oikos_Citizens-Energy_Print.pdf (2019).

Bulgaria (BGR)

New forms of citizen-led energy initiatives are in its infancy in Bulgaria. We identify one energy community that just started (Izgrei 2022). Legal forms for cooperatives and social benefit associations exist in Bulgaria. The government is making progress on the implementation of EU Directives by removing financial and legal barriers for citizens to engage in the energy transition and form energy communities (European Commission 2020), but the topics of individual and collective prosumption are only emerging (Vladimirov, Georgiev and Kolarova (2018), Todorović (2020) and (2021), Ministry of Energy Ministry of the Environment and Water (2019)). Alleviation of energy poverty and energy security are important motivations for enabling community energy initiatives in the country. An interesting example is the attempt to explore options for forming a cross-border cooperation between Burgas in Bulgaria and Nis in Serbia to co-own PV systems. This project mPower is financed by the Horizon 2020 Program of the European Union.

Contributors

Valeria Jana Schwanitz, Ingrid Knutsdotter Koren

References

European Commission. *Commission staff working document, Assessment of the final national energy and climate plan of Bulgaria*. SWD (2020) 901 final. https://ec.europa.eu/energy/sites/default/files/documents/staff_working_document_assessment_necp_bulgaria_en.pdf (2020).

Izgrei. All electricity suppliers in one place. https://www.izgrei.bg/ (2022).

Ministry of Energy and Ministry of the Environment and Water. *Integrated energy and climate plan of the Republic of Bulgaria 2021-2030*. Republic of Bulgaria. https://ec.europa.eu/energy/sites/default/files/documents/bg_final_necp_main_en.pdf (2019).

Todorović, I. Bulgarian municipalities advised on possibilities for energy communities. *Balkan Green energy news*. https://balkangreenenergynews.com/bulgarian-municipalities-advised-on-possibilities-for-energy-communities/ (2020).

Todorović, I. Niš is empowering citizens within its energy transition. *Balkan Green energy news.* https://balkangreenenergynews.com/nis-is-empowering-citizens-within-its-energy-transition/ (2021).

Vladimirov, M., Georgiev, A. & Kolarova, S. Development of small-scale renewable energy sources in Bulgaria: Legislative and administrative challenges. *Center for the study of democracy*. https://csd.bg/publications/publication/development-of-small-scale-renewable-energy-sources-in-bulgaria-legislative-and-administrative-chal/ (2018).

Croatia (HRV)

Methodological approach

A small number of cooperatives was identified based on two main sources. These are, first, a presentation given by Edo Jerkić, a member of one of the larger energy cooperatives in Croatia, named Zelena energetska zadruga (HRV_1_R) and, second, a list of energy cooperatives available on the National Energy Efficiency Portal (HRV_2_O). Additional administrative information (register number, address, etc.) was then collected from the official Croatian business register (HRV_3_OR). Information on production units was occasionally available on individual websites, and newspaper articles (HRV_4_O) as well as in HRV_1_R.

Validation & completeness

Data accuracy: Where possible, information from the report by Edo Jerkić (HRV_1_R), the National Energy Efficiency Portal (HRV_2_O), the business register (HRV_3_OR), and individual websites and media (HRV_4_O) was cross-checked. A follow-up interview with Edo Jerkić was conducted in 2019. **Data collection errors:** Four eyes principle, automated check for duplicates and wrong data formats during generation of data implementation files. **Validation of aggregates/Representativeness of sample:** Comparison with previous studies: Jerkić (2016) lists 7 initiatives; Kirac (2018) mentions a total of 9 initiatives; Eichermuller (2018) mentions a total of 5 initiatives and estimates the number of people involved at around 100. The follow-up interview with Edo Jerkić in 2019 was further used to validate our dataset.

Contributors

Jan Pedro Zeiss, Ingrid Knutsdotter Koren, Valeria Jana Schwanitz

References

HRV_1_R, Edo Jerkić, Energy cooperatives in Croatia, https://www.irena.org/-/media/Files/IRENA/Agency/Events/2016/Jan/18/ZEZ-Energy-coops-in-Croatia.pdf?

HRV_2_O, Nacionalni portal energetske učinkovitosti. Energetske zadruge (List of energy cooperatives on Website of National Energy Efficiency Portal), https://www.enu.hr/ee-u-hrvatskoj/tko-je-tko-ee-rh/energetske-zadruge

HRV_3_OR, Official Croatian business register, https://sudreg.pravosudje.hr/registar/f?p=150:1

HRV_4_O, Initiatives' websites, (social) media, n/a

Additional references

Eichermüller, J., Furlan, M., Habersbrunner, K. & Kordić, Z. Energy cooperatives - Comparative analysis in Eastern partnership countries and Western Balkans. *Women engage for a common future*. http://www.wecf.org/wp-content/uploads/2018/06/EnergyCoops_LongOnline.pdf (2018).

Jerkic, E. Energy coops in Croatia. *Zelena energetska zadruga*. https://www.irena.org/-/media/Files/IRENA/Agency/Events/2016/Jan/18/ZEZ-Energy-coops-in-Croatia.pdf?la=en&hash=E72E78396642F36E972B9EE840CD09745A419746 (2016).

Kirac, M., Furlan M. & Pašičko, R. Green power to the people – Croatian perspective on community energy. *Heinrich Böll Stiftung Belgrade*. https://rs.boell.org/en/2020/04/10/green- power-people-croatian-perspective-community-energy (2018).

Cyprus (CYP)

Methodological approach

No data was collected for Cyprus as there are no relevant initiatives active yet and the topics of individual and collective prosumption as well as energy communities are only emerging (CEA 2021, EAC 2021). According to the Cyprus Energy Agency (CEA), it is expected that the first energy communities will soon be able to be established in Cyprus, supporting the process of building up knowledge with guidelines and manuals. The energy system is still centralized with 100% of the electricity generation being owned by the Electricity Authority of Cyprus (EAC). The government is making progress on the implementation of EU Directives that target the removal of financial and legal barriers for citizens to engage in the energy transition (European Commission 2020).

Validation & completeness

Not applicable.

Contributors

Ingrid Knutsdotter Koren, Valeria Jana Schwanitz

References

Cyprus Energy Authority (CEA). *Energy communities*. https://www.cea.org.cy/en/energeiakes-koinotites (2021).

Electricity Authority of Cyprus (EAC). *Non regulated activities*. https://www.eac.com.cy/EN/NonRegulatedActivities/about/Pages/default.aspx (2021).

European Commission. *Commission staff working document, Assessment of the final national energy and climate plan of Cyprus*. SWD (2020) 912 final. https://ec.europa.eu/energy/sites/default/files/documents/staff_working_document_assessment_necp_cyprus_en.pdf (2020).

Czech Republic (CZE)

Methodological approach

Cooperatives were primarily identified in The Czech Energy Atlas (CZE_1_DB) through the search term "Družstvo" (cooperative). This source provided information on owner, installed capacity, technology, and device specifications of each production unit. Then, the identified owners were searched in the Czech Business Register (CZE_2_OR) where information on the registered name, ID, address, yearly number of shares, year of foundation and cancellation, and activity was extracted.

Validation & completeness

Data accuracy: No cross-referencing possible, as only one data source for each data record exists. **Data collection errors:** Four eyes principle, automated check for duplicates and wrong data formats during generation of data implementation files. **Validation of aggregates/Representativeness of sample:** Maly (2019) states that cooperatives own 1% of total installed renewable energy capacity and 23% of total biomass/biogas capacity. In comparison, the units included in the inventory represent 2% of the total capacity or 7% of the biomass/biogas capacity of the Czech Energy Atlas (CZE_1_DB). **Further remarks:** According to "Law No. 513/1991 of Coll., the Commercial Code ('Obchodní zákoník')" all cooperatives in the Czech republic are legally obliged to include the term "Družstvo" in their name. We therefore assume that a search for the term "Družstvo" in CZE_1_DB should result in a comprehensive list of cooperatives that own energy production units. However, evidence from other literature estimates a higher number of cooperatives than we were able to identify (see ‘Current total number of initiatives’). It is possible the Czech energy atlas (CZE_1_DB) is not complete, which would consequently result in an under-representativeness of our data as well.

Contributors

Jan Pedro Zeiss, Valeria Jana Schwanitz, Ingrid Knutsdotter Koren

References

CZE_1_DB, Czech Energy Atlas, http://www.calla.cz/atlas/index.php

CZE_2_OR, Official Czech business register, https://or.justice.cz/ias/ui/rejstrik

Additional references

Malý, V., Šafařík, M. & Matoušek, R. Consumer (co-)ownership in renewables in the Czech Republic. *Energy transition financing consumer co-ownership in renewables*, J. Lowitsch (Ed.), Palgrave Macmillan: Cham, 201-222. https://doi.org/10.1007/978-3-319-93518-8 (2019).

Denmark (DNK)

Methodological approach

The identification of Danish citizen-led initiatives is challenging as dedicated legal forms are lacking. For example, laws regulating the form of cooperatives do not exist. While many of the registered initiatives show some elements of cooperative principles in their governance structure, they are mostly registered as partnerships (interessentskab). Typically, cooperative governance structures are agreed upon in the bylaws. To distinguish between partnerships with a cooperative focus and those without, the following approach has been taken: the Danish business register is the primary source of information (DNK_1_OR). The registry has been screened for partnerships (interessentskab (I/S)) engaging in the relevant economic sectors (namely, 351300 Distribution af elektricitet, 351100 Produktion af elektricitet, 351400 Handel med elektricitet, and 351200 Transmission af elektricitet). The registration numbers (cvr ID) were used to obtain ownership information about the partnership. Only those partnerships which have more than 10 founding members and are still active as of 12/2021 were selected. It was outside of the scope to analyze the bylaws (vedtægter) of approximately 1000 initiatives. Instead, those entities that include the following statement were added to the inventory: The real owner cannot be identified or found, the board is used as the real owner ("Reel ejer kan ikke identificeres eller findes ikke, ledelsen indsat som reel ejer"). This statement indicates that no majority shareholder exists and hence, it was taken as a proxy. Proceeding this way, 1125 partnerships are or have been active in the economic sector 351100 "Production of electricity". Out of these 1125 partnerships, 542 are still active as of 2021-09-30. After screening for ownership, a sample of 117 partnerships was used to check information on the initiatives' websites (DNK_3_O).

Information on the production units in Denmark on wind was collected through the Danish Energy Agency (DNK_2_OR), which is largely restricted from public access. We were able to access the data provided by the Danish Technical University, but matching between initiatives and production units has to be kept anonymous due to data regulations. Therefore, we list all units with information on their capacity and location, but without revealing ownership information. Most of the district heating suppliers in Denmark are registered as partnership with limited liability or as association ("Andelsselskab med begrænset ansvar" (Amba), "Forening"). However, being registered in this way does not imply a direct influence of citizens on the decisions of the company. To verify a collective engagement, the following steps have been taken: 1) ownership information on DNK_1_OR such as "Beneficial owners cannot be identified, senior management is registered as beneficial owners" and 2) checking the website for keywords such as "forbrugerejet fjernvarmeværk". In a few cases, the bylaws have been checked to verify the perception of collective ownership obtained from steps 1) and 2). Information on solar thermal

installations in district heating plants was sourced from the website. If information on year of installation or expected yearly generation was not available on this site, an additional internet search was performed to complete information where possible. Following this selection of initiatives, production units were taken from the Danish Energy Agency (DNK_3_OR). Contrary to wind energy production units, this information is publically available.

Validation & completeness

Data accuracy: No cross-referencing possible, as only one datasource for each data record exists. **Data collection errors:** Four eyes principle, automated check for duplicates and wrong data formats during generation of data implementation files. **Validation of aggregates/Representativeness of sample:** Comparison with Gorroño, Sperling, Djørup (2019).

Contributors

August Wierling, Jan Pedro Zeiss, Lukas Müller, Ingrid Knutsdotter Koren, Heather Arghandeh Paudler, Valeria Jana Schwanitz

References

DNK_1_OR, Danish business register, https://datacvr.virk.dk/data/?language=en-gb

DNK_2_OR, Danish Energy Agency, internal document accessing non-public data provided by the Danish Technical University

DNK_3_O, Initiatives' websites, (social) media, n/a

DNK_4_OR, Danish Energy Agency, Energy Statistics, https://ens.dk/en/our-services/statistics-data-key-figures-and-energy-maps/energy-statistics-definitions-and-methods

Additional references

Gorroño-Albizu, L., Sperling, K. & Djørup, S. The past, present and uncertain future of community energy in Denmark: Critically reviewing and conceptualising citizen ownership. *Energy research & social science*. 57, 101231; https://doi.org/10.1016/j.erss.2019.101231 (2019).

Mey, F.; Diesendorf, M. Who owns an energy transition? Strategic action fields and community wind energy in Denmark. *Energy research & social science*. 35, 108–117; https://doi.org/10.1016/j.erss.2017.10.044 (2018).

Wierling, A. *et al*. Statistical evidence on the role of energy cooperatives for the energy transition in European countries. *Sustainability*. 10, 3339; https://doi.org/10.3390/su10093339 (2018).

Estonia (EST)

Methodological approach

The primary identification of initiatives was based on the dataset compiled by the Tartu Regional Energy Agency (EST_1_DB), which itself was compiled through a survey and direct contact with initiatives. Data on energy generation units was extracted from solar system design firms' websites (EST_5_O, EST_6_O, EST_7_O), the initiatives' websites (EST_8_O, EST_9_O) and the

register of buildings (EST_3_OR). Financial data was gathered from the e-Business Register (EST_2_OR).

Validation & completeness

Data accuracy: Where possible, cross-referencing of information from TREA dataset (EST_1) with initiatives' websites (EST_8, EST_9) and business and buildings registers (EST_3, EST_4) and further verification of initiatives in Estonian Land Board map application (EST_4). **Data collection errors:** Four eyes principle, automated check for duplicates and wrong data formats during generation of data implementation files. **Validation of aggregates/Representativeness of sample:** Data on citizen-led initiatives in Estonia are not reported in other literature. For example, REScoop.eu does not list any initiative for the country. Our data are sourced from official registries cross-checked with country experts and other sources of information, which is why we have confidence in the data. The implementation of the EU Directives is completed and necessary regulations are amended. However, Estonia does not strongly emphasize citizen-participation in its interpretation of the concept "energy community". This matches the high degree of centralization of the Estonian energy system.

Contributors

Grete Glaase, Valeria Jana Schwanitz, Jan Pedo Zeiss, Lukas Müller

References

EST_1_DB, TREA (Tartu Regional Energy Agency) - Dataset on energy communities compiled by TREA in the context of the COMETS project

EST_2_OR, e-Business Register, https://ariregister.rik.ee/

EST_3_OR, Register of Buildings, https://livekluster.ehr.ee/ui/ehr/v1

EST_4_OR, Estonian Land Board map application, https://xgis.maaamet.ee/xgis2/page/app/maainfo

EST_5_O, Solar system design firm homepages, https://energiapartner.ee/

EST_6_O, Solar system design firm homepages, http://napssolar.ee

EST_7_O, Solar system design firm homepages, https://solar4you.ee/

EST_8_O, Initiatives' websites, (social) media, n/a

EST_9_O, Project homepages, n/a

Finland (FIN)

Methodological approach

Data were sourced from official registries and cooperatives' websites. Identified initiatives in the searches were manually checked for their engagement in activities related to the energy transition. The search terms "energ" and "sähkö" (electricity) were used in the Finish business register (FIN_1_OR) and filtered by the legal form "cooperative" (osuuskunta). All results that

could be identified as renewable energy cooperatives (either through the name directly or through websites, if available) were included in the database. Cooperatives listed under Finish industrial sector codes 35, 49 - 53, 855, 856 (Statistics Finland, 2008) were searched for in Asiakastieto (FIN_3_DB). The Business Register operated by a Finish financial newspaper (FIN_5_DB) was used to search for cooperatives listed under Finish industrial sectors codes 35, 37, 3821, 46120, 46711, 46719, 4931, and 85599. Basic administrative information was extracted from FIN_1_OR-FIN_6_O, and annual turnover was extracted from FIN_3_DB. Additional administrative and financial information and information on energy generation units was extracted from initiatives' websites (FIN_6_O).

Validation & completeness

Data accuracy: Cross-referencing of information from different business registers and websites. **Data collection errors:** Four eyes principle, automated check for duplicates and wrong data formats during generation of data implementation files. **Validation of aggregates/Representativeness of sample:** Letonen 2019 reported that in 2015, 12.7% of the total 618 heat entrepreneurs were cooperatives. This amounts to 78 heating cooperatives. Huhtala 2016 estimated 7 "first wave" energy cooperatives and 120 "new wave" energy cooperatives. Ruggiero 2019 informs about 2 wind power cooperatives. **Further remarks:** Step 3 of the data collection process from 2019 was not repeatable in 2021, as the systematic search option along industrial sector classifications in FIN_5 could not be found anymore. A similar approach is possible using the Open Corporates business register (FIN_7). However, here it is only possible to select the main industrial sector categories (two digit categories) and not the sub-categories. As category 35 "Electricity, gas, steam and air conditioning supply" is the only main category that is relevant in its entirety, it was only possible to cross-reference initiatives in this category. This resulted in a total of 57 initiatives. 50 of our previously identified initiatives could be matched in this process. 7 New initiatives were added.

Contributors

Jan Pedro Zeiss, Constantin von Beck, Ingrid Knutsdotter Koren

References

FIN_1_OR, VIRRE - Finnish Patent and Registration Office, https://virre.prh.fi

FIN_2_OR, YHDISTYSREKISTERI - Finnish Patent and Registration Office, Finnish Association Register, https://yhdistysrekisteri.prh.fi

FIN_3_DB, ASIAKASTIETO - Commercial business register, https://www.asiakastieto.fi

FIN_4_DB, FINDER - Finnish "Yellow Pages", https://www.finder.fi/

FIN_5_DB, TALOUSSANOMAT - Finnish financial newspaper, https://www.is.fi

FIN_6_O, Initiatives' websites, (social) media, n/a

FIN_7_DB, Open Corporates Register, https://opencorporates.com/

Additional references

Huhtala, K. *Cooperatives in Finland –What they are like and how they operate*. https://www.chrissmithonline.co.uk/files/kari_edinburgh_2_2016.pdf (2016).

Lehtonen, O., & Okkonen, L. Energy cost reduction creates additional socioeconomic benefits– The case of Eno Energy Cooperative, Finland. *Energy Policy*. 129, 352–359; https://doi.org/10.1016/j.enpol.2019.02.018 (2019).

Ruggiero, S., Isakovic, A., Busch, H., Auvinen, K. & Faller, F. *Developing a joint perspective on community energy: Best practices and challenges in the Baltic Sea region*. Co2mmunity project, Working paper No. 2.3. http://co2mmunity.eu/wp-content/uploads/2019/03/Co2mmunity-working-paper-2.3.pdf (2019).

Statistics Finland. *Standard Industrial Classification TOL 2008*. https://www.stat.fi/en/luokitukset/toimiala/ (2008).

France (FRA)

Methodological approach

Initial prospecting was done primarily through the Energie Partagée website, map tool, and database (FRA_2_DB) and through the Centrales Villageoises database export (FRA_3_DB) which was provided by that organization. All initiative websites and other internet presences (FRA_1_O) were reviewed, when such existed, and further initiatives were identified by snowballing from these information sources. Administrative and some financial information was gathered from websites which report from official registries, such as the Figaro (FRA_4_OR) and Societe.com (FRA_5_OR) websites, and if the initiatives in question were associations, from the government journal of associations (FRA_6_OR) and net1901 websites (FRA_7_DB). Detailed organizational information for initiatives was gathered from initiative websites (FRA_1_O). Information on production units was mainly gathered from initiative websites (FRA_1_O), the Energie Partagée website (FRA_2_DB), online production tracking tools (FRA_9_O), and, in the case of wind-power units, The Wind Power database (FRA_10_DB). Additional units identified and information gathered using Enercoop's producer map (FRA_11_DB). Geolocation information for units was occasionally found on initiative websites (FRA_1_O), but more often from Google Maps (FRA_8_O) by using satellite imagery to confirm exact locations of installations referenced on initiative websites.

Validation & completeness

Data accuracy: cross-referencing data with existing datasets, in particular Energie Partagée and Centrales Villageoises. **Data collection errors:** Four eyes principle, automated check for duplicates and wrong data formats during generation of data implementation files. **Validation of aggregates/Representativeness of sample:** Comparison with previous studies (generally out of date), and aggregates listed in Energie Partagée database. **Further remarks:** The perimeter for inclusion in France is more complex than most countries because of the large variety of legal forms existent in the country, and the number of those that are accepted by local actors as potentially being a part of the "citizen energy" movement. The choices made for which initiatives to include or exclude are a compromise between the definitions local actors use and other (generally broader) definitions for what constitutes a collective action initiative in the energy transition. In particular, our perimeter of study includes both non-profit associations and mobility-related collective actions, which are not generally reported as a part of "citizen energy" by local actors in France. This means that the aggregates found in the database here will necessarily differ from those reported by other local actors and studies. However, these general

aggregates match relatively closely with the figures reported in other sources, leading to a high level of confidence in the accuracy of the present dataset.

ADEME, (2016). Quelle intégration territoriale des énergies renouvelables participatives? (Initiatives Identified = 165), Energie Partagée database (2021) (Initiatives identified = 283)

Contributors

Timothy Marcroft, August Wierling

References

FRA_1_O, Initiatives' websites, (social) media, n/a

FRA_2_DB, Energie Partagée map, https://energie-partagee.org/decouvrir/energie-citoyenne/tous-les-projets/

FRA_3_DB, Centrales Villageoises data export (May 2020), not available online

FRA_4_OR, Le Figaro business registry portal, https://entreprises.lefigaro.fr/

FRA_5_OR, Societe.com business registry portal, https://www.societe.com/

FRA_6_OR, Official Journal of associations, https://www.journal-officiel.gouv.fr/associations/

FRA_7_DB, Net1901 association database, http://www.net1901.org/

FRA_8_O, Google Maps, https://www.google.com/maps

FRA_9_O, Online production tracking tools, n/a

FRA_10_DB, The Wind Power database, https://www.thewindpower.net/index.php

FRA_11_DB, Enercoop producer map, https://www.enercoop.fr/la-production-de-notre-electricite/nos-producteurs

Germany (DEU)

Methodological approach

The German data set comprises mainly cooperatives ("eingetragene Genossenschaft - e.G") and associations ("eingetragener Verein - e.V"). Individual initiatives with other legal forms were included if specifically identified as relevant.

Cooperatives: An initial sample was generated through a keyword search in German business registers (GER_1_OR, GER_2_OR, GER_3_OR, GER_4_DB; keywords: Bürgerenergie, Energiegenossenschaft, erneuerbare Energie, Bürgerwerke, Bürgerwind, Bürgersolarpark). A second systematic search for relevant cooperatives was conducted in the German Core Energy Market Data Register (GER_1_OR). This new register contains data on all renewable energy production facilities installed in Germany. The deadline for registration of existing units was 31.01.2021. A search within this register was conducted in 2021 and repeated in 2022, after the registration deadline, by searching for the terms "eg", "e.g" and "genossenschaft" in the name of the owner of the production unit. Further initiatives were identified through snowball sampling

of mentioned initiatives on websites of already identified cooperatives (GER_7_O), as well as from various other sources such as newspaper articles and social media. Basic administrative data was collected from German business registers (GER_2_OR - GER_6_OR). Financial data and data on membership evolution were sourced from annual financial reports published in the Unternehmensregister (GER_6_OR). If annual financial reports were not available, data was collected from individual initiatives' websites (GER_7_O). Data on energy production facilities was gathered from the German Core Energy Market Data Registry (GER_1_OR) and individual initiatives' websites (GER_7_O).

Associations: A market data registry search (GER_1_OR) for e.V. (abbrev. for registered association) in the name of the renewable production unit owners generated the initial sample of energy producing associations. This initial sample was complemented by a keyword search in the official association register (GER_3_OR) for the keywords "energie", "solar", "wind", "wärme" or "erneuerbar" in the name field of registered names. In the further process, both keyword searches were merged by names to connect production units and their owners. In addition, corresponding websites of associations were checked for information and used as a starting point for further snowball sampling.

Mobility Initiatives: The separate German data set with mobility-related initiatives comprises two main activities: electric vehicle charging as well as operating local car sharing systems with electric cars (Cooperative e-car sharing initiatives, CeCSI). The legal form of the initiatives is mainly cooperatives ("eingetragene Genossenschaft - e.G") and associations ("eingetragener Verein - e.V").

For the first type of activities, a systematic search for cooperatives operating car charging stations was conducted in the German car charging stations registry ("Ladesäulenregister", GER_9_OR), which is maintained by the German Association of Energy and Water Industries ("BDEW Bundesverband der Energie- und Wasserwirtschaft e.V.") on behalf of the Federal Network Agency ("Bundesnetzagentur"). It is mandatory to report public car charging stations to the German car charging stations directory after putting them into operation. A search within a snapshot of this register as of Jan 1st, 2022, was conducted by searching for the terms "eg", "e.g" and "genossenschaft" in the operator name data field of the charging station, followed by a download of the resulting entries.

For the first type of activities, the initiatives were identified through snowball sampling of mentioned initiatives on websites of already identified cooperatives (GER_7_O), as well as from various other sources such as newspaper articles and social media. Basic administrative data was collected from German business registers (GER_2_OR - GER_6_OR). Data about the number of shared cars, the development characteristics of the car sharing network and about operation details were gathered from the web sources mentioned above, complemented by additional information through interviews with representatives of the initiatives.

The specialized dataset containing mobility-related initiatives contains, beside basic administrational data, also some contextual data for mobility-related activities, about units like shared cars and car charging stations. These data are partly aggregated and categorized.

Validation & completeness

Data accuracy: Cross-referencing of information among different business registers and websites. **Data collection errors:** Four eyes principle, automated check for duplicates and wrong data formats during generation of data implementation files. Selected PV units within the association sample were double checked, when the reported number of modules was not in line

with currently available module capacities. However, we expect the reported capacities to be correct and implimented the data as displayed in GER_1_OR. **Validation of aggregates/Representativeness of sample:** Comparison with previous studies and information provided by other actors, such as renewable energy traders focusing on selling energy from citizen energy initiatives (Bürgerwerke, Bavaria-Strom) or the German Cooperative Association (Deutscher Genossenschafts- und Raiffeisenverband). **Further remarks:** For the dataset cogeneration units were only included when they are operated with a renewable fuel source.

Contributors

Associations: Constantin von Beck, Lukas Müller; Mobility initiatives: Tobias Kraudzun, August Wierling; All other: August Wierling, Constantin von Beck, Tobias Kraudzun, Jan Pedro Zeiss, Simon Dufner, Heather Arghandeh Paudler, Lukas Müller.

References

GER_1_OR, Core Energy Market Data Registry, https://www.marktstammdatenregister.de

GER_2_OR, Online trade registry, https://www.online-handelsregister.de

GER_3_OR, Trade registry, https://www.handelsregister.de

GER_4_DB, Northdata, https://www.northdata.com/

GER_5_DB, Cylex, https://web2.cylex.de/

GER_6_OR, Unternehmensregister, https://www.unternehmensregister.de

GER_7_O, Initiatives' websites, (social) media, n/a

GER_8_R, Annual financial accounts, https://www.unternehmensregister.de

GER_9_OR, German car charging stations registry, https://ladesaeulenregister.de/

Additional references

Bauknecht, D., Funcke, S., & Vogel, M. Is small beautiful? A framework for assessing decentralised electricity systems. *Renewable and sustainable energy reviews*. 118, 109543; https://doi.org/10.1016/j.rser.2019.109543 (2020).

Broska, L. H. It's all about community: On the interplay of social capital, social needs, and environmental concern in sustainable community action. *Energy research & social science*. 79, 102165; https://doi.org/10.1016/j.erss.2021.102165 (2021).

Centgraf, S. Supporting civic engagement in German energy cooperatives – Transdisciplinary research based on the reflection of individual needs. *Energy research & social science*. 44, 112; https://doi.org/10.1016/j.erss.2018.05.003 (2018).

Herbes, C., Rilling, B., & Holstenkamp, L. Ready for new business models? Human and social capital in the management of renewable energy cooperatives in Germany. *Energy policy*. 156, 112417; https://doi.org/10.1016/j.enpol.2021.112417 (2021).

Schmid, B., Meister, T., Klagge, B., & Seidl, I. Energy cooperatives and municipalities in local energy governance arrangements in Switzerland and Germany. *The Journal of environment and development*. 29/1; https://doi.org/10.1177%2F1070496519886013 (2019).

Schwabe, J. The evolution of cooperative electric carsharing in Germany and the role of intermediaries. *Environmental innovation and societal transitions*. 37; https://doi.org/10.1016/j.eist.2020.08.007 (2020).

Süsser, D. *et al*. Harvesting energy: Place and local entrepreneurship in community-based renewable energy transition. *Energy policy.* 101, 332; https://doi.org/10.1016/j.enpol.2016.10.018 (2017).

Wierling, A., Zeiss, J.P., Lupi, V., Candelise, C., Sciullo, A. & Schwanitz, V.J. The contribution of energy communities to the upscaling of photovoltaics in Germany and Italy. *Energies*. 14/8, 2258; https://doi.org/10.3390/en1408225 (2021).

Great Britain (GBR)

Methodological approach

Identification of initiatives began with the database downloaded from Cooperatives UK (GBR_1_OR) and filtered using the British industrial sector code "35100" which represents "Electric power generation, transmission and distribution." This database was then cross-referenced with the Financial Conduct Authority database (GBR_2_OR) which has all organizations registered under the "Co-operative and Community Benefits Society Act 2014". Basic administrative data was collected from GBR_1_OR and GBR_2_OR. Financial data was collected from GBR_2_OR. Data related to energy production was collected from annual reports downloaded from GBR_2_OR and from initiatives' websites and (social) media (GBR_3_O). NACE code 35100, Electric power generation, transmission and distribution was used to search for relevant activities.

Validation & completeness

Data accuracy: cross-checking of information with previous study GBR_4_P followed by checking on organizations' websites, social media pages, publicly available documents and media articles found via Google searches. **Data collection errors:** Four eyes principle, automated check for duplicates and wrong data formats during generation of data implementation files. **Validation of aggregates/Representativeness of sample:** Comparison with previous studies. Cross-referencing of initiatives identified from GBR_4_P revealed 31 newly established initiatives from 2019 through 2020, and 14 were dissolved during the same time frame. **Further remarks:** If organizations in the Cooperatives UK (GBR_1) did not exist in Financial Conduct Authority (GBR_2) they were flagged but not removed from the database (this was the case for 10 initiatives).

Contributors

August Wierling, Melake Getabecha, Philippa Roots, Mehran Ziyabadi

References

GBR_1_OR, Cooperatives UK, https://www.uk.coop/

GBR_2_OR, Financial Conduct Authority, https://mutuals.fca.org.uk/

GBR_3_O, Initiatives' websites, (social) media, various sources

GBR_4_P, Wierling *et al*. (2018), https://doi.org/10.3390/su10093339

Greece (GRC)

Methodological approach

Initial identification of initiatives was based on a map of energy communities constructed by Greenpeace (GRC_1_R_DB). Then a search for entries from the initial step was conducted in Greek business registers (GRC_2_OR - GRC_5_DB). If entries could be found in business registers, they were added to the inventory. Administrative and financial data was gathered from GRC_2_OR - GRC_4_OR. Data on energy generation was gathered from GRC_6_DB and GRC_7_O.

Validation & completeness

Data accuracy: An open point about existing energy communities in Greece is the question to what extent the initiatives are truly citizen-led and not a vehicle for private companies to benefit from governmental support for energy communities (Tsagkari 2020, Douvitsa 2018). We note this discussion but have no ground to exclude individual initiatives. A revision is subject to future research. **Data collection errors:** Four eyes principle, automated check for duplicates and wrong data formats during generation of data implementation files. **Validation of aggregates/Representativeness of sample:** Comparison with previous studies. Vasilakis 2020 reports 409 initiatives and Frieden 2020 reports 300. We therefore cover approximately 50% of initiatives in comparison to other publications.

Contributors

Ingrid Knutsdotter Koren, Zacharias Andreadakis, Valeria Jana Schwanitz

References

GRC_1_R_DB, Greenpeace - online map of energy communities, https://www.google.com/maps/d/u/0/viewer?mid=1lb0zwm5fQACnQbmlUU_nPxjd2YwEMIVN&ll=35.31551653008427%2C25.15266586278034&z=13

GRC_2_OR, GEMI - business register of Greece, https://www.businessregistry.gr/publicity/index

GRC_3_OR, Athens Chamber of Commerce and Industry, https://directory.acci.gr/companies

GRC_4_OR, eChamber, https://echamber.veth.gov.gr/eChamber/login.php?action=sSearchEponimia

GRC_5_DB, Findbiz B2B, https://findbiz.gr/en/about-us/

GRC_6_DB, MES Energy website list of energy community units, https://enia.online/en/companies/

GRC_7_O, Initiatives' websites, (social) media, n/a

Additional references

Douvitsa, I. The New Law on Energy Communities in Greece. *Cooperativismo e economía social*. 40; https://doi.org/10.35869/ces.v0i40.1385 (2018).

Frieden, D. & Tuerk, A. *Deliverable 2.3: Regulatory frameworks for energy communities in the pilot site countries Croatia, Spain, Greece, Portugal and Slovenia, Shaping EU framework transposition and project implementation*. Compile project, Deliverable 2.3. https://www.researchgate.net/publication/346652927_Deliverable_23_Regulatory_frameworks_for_energy_communities_in_the_pilot_site_countries_Croatia_Spain_Greece_Portugal_and_Slovenia_Shaping_EU_framework_transposition_and_project_implementation (2020).

Tsagkari, M. How Greece undermined the idea of renewable energy communities: An overview of the relevant legislation. *Law environment and development journal (LEAD)*. 17; https://www.researchgate.net/publication/346096106_How_Greece_Undermined_the_Idea_of_Renewable_Energy_Communities_An_Overview_of_the_Relevant_Legislation (2020).

Vasilakis, A. *et al*. *Mapping of Energy Communities in Greece*. HP1AB-00256, Deliverable 1.1. https://www.greenpeace.org/static/planet4-greece-stateless/184045bd-mapping_of_energy_communities_v1.2.pdf (2020).

Hungary (HUN)

Methodological approach

The concept of community energy as well as similar forms have not formally been established yet in Hungary. A few small-scale projects have grown from existing community structures (Capellan-Perez 2020), but further information is missing except for one of these initiatives. We therefore only report this one initiative. Overall legal and financial barriers to collective action are high and individual prosumers are prefered (European Commission 2020).

Validation & completeness

Data accuracy: Not available. **Data collection errors/Validation:** Four eyes principle, automated check for duplicates and wrong data formats during generation of data implementation files.

Contributors

Constantin von Beck, Ingrid Knutsdotter Koren

References

HUN_1_O, Friends of Earth, https://friendsoftheearth.eu/wp-content/uploads/2020/01/community_energy_in_hungary_and_czechia_briefing.pdf

HUN_2_O, Golya Cooperative, http://golyapresszo.hu/golya-2/story/

Additional references

European Commission. *Commission staff working document, Assessment of the final national energy and climate plan of Hungary*. SWD (2020) 916 final. https://ec.europa.eu/energy/sites/default/files/documents/staff_working_document_assessment_necp_hungary_en.pdf (2020).

Ireland (IRL)

Methodological approach

No standardized data sources allowing systematic search for renewable energy initiatives were available for Ireland. A list of 14 initiatives was compiled based on information from various sources, such as the map of 5 energy cooperatives provided by Energy Cooperatives Ireland (IRE_1_O) and individual websites (IRE_3_O). The official Irish business register (IRE_2_OR) does not allow searching for specific legal forms or industrial activities. Basic administrative data for the identified initiatives was gathered from the IRE_2_OR. Additional information on energy production units was collected from the initiatives' individual websites (IRE_3_O). In addition, the Sustainable Energy Authority of Ireland hosts a list of ca. 300 Sustainable Energy Communities (SEAI, 2017b). However, due to a lack of clear defining criteria for these Sustainable Energy Communities, they have not been included in the inventory.

Validation & completeness

Data accuracy: Administrative data was collected from the official business register, ensuring data accuracy. Further systematic cross-checking of data between several sources was not possible. **Data collection errors:** Four eyes principle, automated check for duplicates and wrong data formats during generation of data implementation files. **Validation of aggregates/Representativeness of sample:** While cooperatives in general are well established in Ireland (see for instance ICOS 2021), energy cooperatives are still in their early stages. This is especially true for energy-producing cooperatives. A number of energy cooperatives exist in Ireland, however they seem mostly focused on awareness raising, consulting work, and energy efficiency improvements for their members (SEAI 2017a). Several sources state that in 2016, there was only one electricity producing wind farm, the Templederry Community Wind Farm (See Community Power 2021, Thompson 2016). According to Community Power (2021) the lack of electricity producing cooperatives is caused by the difficulties of connecting to the grid. We have concluded that we have satisfactory coverage of the current state of activities in the country.

Contributors

Jan Pedro Zeiss, Philippa Roots, Valeria Jana Schwanitz, Heather Arghandeh Paudler, Constantin von Beck, Shirin Mohammadi, Tim Marcroft, Lukas Müller

References

IRL_1_O, Energy Co-operatives Ireland Ltd, Co-ops Maps, https://www.energyco-ops.ie/setting-up-a-co-op/co-ops-map/

IRL_2_OR, Companies Registration Office, CORE Business Register, https://core.cro.ie/search

IRL_3_O, Initiatives' websites, (social) media, n/a

Additional references

Community Power. *Ireland*. https://www.communitypower.eu/en/ireland.html (2021).

The Irish Co-operative Organization Society (ICOS). *Map of Co-operatives.* http://icos.ie/find-your-co-op/ (2021).

Thompson, S. Power to the people: is community energy the way forward? *The Irish Times*; https://www.irishtimes.com/news/environment/power-to-the-people-is-community-energy-the-way-forward-1.2515437 (2016).

Sustainable Energy Authority of Ireland (SEAI). *Energy Communities*. https://www.seai.ie/community-energy/sustainable-energy-communities/ (2022).

Sustainable Energy Authority of Ireland (SEAI). *Search our Sustainable Energy Community Network*. https://www.seai.ie/community-energy/sustainable-energy-communities/sec-map/index.xml (2022).

Italy (ITA)

Methodological approach

The Italian sample has been constructed by merging data from different sources. An initial dataset of 17 initiatives with their core information was provided by Candelise and Ruggieri (ITA_1_P). This dataset was substantially expanded in the following way: Initiatives active in southern Tyrol (many of them historical electric and heating cooperatives) have been identified through Google searches leading to documents published by the regional governments (ITA_2_OR) and the Südtiroler Energieverband (ITA_3_R). The data obtained were cross-checked with the following three sources: The Chamber of Commerce Database for Italy (to check for correct legal forms and national identifiers, ITA_4_DB), the Italian Regulatory Authority for Energy, Networks and Environment (to check purposes and economic classification codes, ITA_5_DB), scientific publications (ITA_6_P, ITA_7_P), and an online map for energy communities (ITA_10_O). In addition, the entries were checked for duplicates using the Italian and German language registries (ITA_8_DB). Production data (i.e., the number and location of PV units, investment costs, and installed capacities) were collected from different websites (ITA_9_O). Data concerning PV units came from web-based searches, analysis of statutes, annual assembly reports, and direct contacts with founders and members of the ECs.

Validation & completeness

Data accuracy: cross-checking of information on websites, documents publicly available and follow up interviews with founders or members of initiatives. **Data collection errors:** Four eyes principle, automated check for duplicates and wrong data formats during generation of data implementation files. **Validation of aggregates/Representativeness of sample:** Comparison with previous studies (Grignani *et al*. 2021, Silvestre *et al*. 2021, Spinicci 2015).

Contributors

August Wierling, Ingrid Knutsdotter Koren, Timothy Marcroft, Heather Arghandeh Paudler, Tobias Kraudzun, Constantin von Beck, Chiara Candelise, Veronica Lupi, Alessandro Sciullo, Jan Pedro Zeiss, Sona Majidi, Nahid Zoubin, Gayatri Sehdev, Valeria Jana Schwanitz

References

ITA_1_P, Candelise, C. & Ruggieri, G., Status and evolution of the community energy sector in Italy, https://doi.org/10.3390/en13081888

ITA_2_OR, various documents from regional government registries in Southern Tirol.

ITA_3_R, Südtiroler Energieverband, https://www.sev.bz.it/en/south-tyrol-energy-association/1-0.html

ITA_4_DB, Italian Chamber of Commerce Database, https://www.ufficiocamerale.it/

ITA_5_DB, Italian Regulatory Authority for Energy, Networks and Environment, https://www.arera.it/it/index.htm

ITA_6_P De Vidovich, L., Tricarico, L., & Zulianello, M, COMMUNITY ENERGY MAP Una ricognizione delle prime esperienze di comunità energetiche rinnovabili, https://www.researchgate.net/publication/357017651_COMMUNITY_ENERGY_MAP_Una_ricognizione_delle_prime_esperienze_di_comunita_energetiche_rinnovabili (2021)

ITA_7_P Barocco *et al*, Le comunità energetiche in Italia, https://doi.org/10.12910/DOC2020-012

ITA_8_DB, Italian Business Registry, https://italianbusinessregister.it/?gclid=Cj0KCQiA8vSOBhCkARIsAGdp6RRqjEZgSTrsCmEAFEPeP73yd6piMjXxEgHXLobuOqqY8rT8HLObc94aAtn6EALw_wcB

ITA_9_O, Initiatives' websites, (social) media, n/a

ITA_10_O, Online GIS map listing the Italian energy community initiatives through a participatory approach, https://experience.arcgis.com/experience/1d992eb312f942959b55c611dd0ce968

Additional references

Candelise, C. & Ruggieri, G. Status and evolution of the community energy sector in Italy. *Energies*. 13/8, 1888; https://doi.org/10.3390/en13081888 (2020).

Grignani, A., Gozzellino, M., Sciullo, A. & Padovan, D. Community cooperative: A new legal form for enhancing social capital for the development of renewable energy communities in Italy. *Energies*. 14/21, 7029; https://doi.org/10.3390/en14217029 (2021).

Silvestre, M.L.D., Ippolito, M.G., Sanseverino, E.R., Sciumè, G. & Vasile, A. Energy self-consumers and renewable energy communities in Italy: New actors of the electric power systems. *Renewable & sustainable energy reviews*. 151, 111565; https://doi.org/10.1016/j.rser.2021.111565 (2021).

Spinicci, F. Le *Cooperative di Utenza in Italia: Casi di Studio*. European research institute on cooperative and social enterprises (Euricse). https://www.euricse.eu/wp-content/uploads/2015/03/1323957261_n1894.pdf (2011).

Wierling, A., Zeiss, J.P., Lupi, V., Candelise, C., Sciullo, A. and Schwanitz, V.J. The contribution of energy communities to the upscaling of photovoltaics in Germany and Italy. *Energies* 14/8, 2258; https://doi.org/10.3390/en1408225 (2021).

Latvia (LVA)

Methodological approach

We have searched the literature and used Google, yielding to the identification of a number of reports and the identification of the "Database of community energy projects in the BSR" (LVA_1_DB). The latter was screened for all relevant projects. The source also provided basic administrative information as well as data on production capacities. All projects relate to housing associations. In addition, the open data file containing all Latvian business register entries (https://data.gov.lv/dati/lv/organization/ur) was searched for all relevant legal forms and business activities. No entries were found during this process.

Validation & completeness

Data accuracy: Cross-checking of information on websites, documents publicly available. **Data collection errors:** Four eyes principle, automated check for duplicates and wrong data formats during generation of data implementation files. **Validation of aggregates/Representativeness of sample:** We could not identify additional databases. The probability is high that additional housing associations exist, but are not registered. Our data therefore provide a conservative estimate. Comparison with gray literature shows that our data collection and assessment captures the current situation in Latvia sufficiently. The topic of community energy is only emerging and citizens face high financial, social, and organizational barriers. Also, cooperative forms are still confronted with historical prejudices, originating from negative experiences during the socialist era. The implementation of EU Directives is ongoing (European Commission 2020). CO2ommunity 2020 reports that currently energy community projects do not exist, whereas Ruggiero *et al*. 2019 and Laes *et al*. 2021 point to LVA_1_DB and housing associations as being the current relevant form.

Contributors

Valeria Jana Schwanitz, August Wierling, Sona Majidi, Ingrid Knutsdotter Koren

References

LVA_1_DB, Database of community energy projects in the BSR,
http://old.lei.lt/co2mmunity/?s=&category=&location=134&a=true

LVA_2_OR, Lursoft, Latvian business register, Lursoft - uzņēmumu datu bāzes

Additional references

Co2mmunity. Co2mmunity, co-producing and co-financing community energy projects. Co2mmunity RENCOPs summary booklet, V1.0. https://co2mmunity.eu/wp-content/uploads/2020/10/2020_09_30_RENCOP_Booklet.pdf (2020).

Community Power. *Latvia*. https://www.communitypower.eu/en/latvia.html (nd).

European Commission. *Commission staff working document, Assessment of the final national energy and climate plan of Latvia*. SWD (2020) 913 final. https://ec.europa.eu/energy/sites/default/files/documents/staff_working_document_assessment_necp_latvia_en.pdf (2020).

Laes, E. *et al*. *Assessment report of potentials for RES community energy in the target regions*. COME RES project, Deliverable 2.2.
https://come-res.eu/fileadmin/user_upload/Resources/Deliverables/Del_2.2_Assessment_Report_of_Potential.pdf (2021).

Ruggiero, S., Isakovic, A., Busch, H., Auvinen, K. & Faller, F. *Developing a joint perspective on community energy: Best practices and challenges in the Baltic Sea region*. Co2mmunity project, Working paper No. 2.3.
http://co2mmunity.eu/wp-content/uploads/2019/03/Co2mmunity-working-paper-2.3.pdf (2019).

Lithuania (LTU)

Methodological approach

We collected the data from the Database of Community Energy Projects in BSR (LTU_1_DB), where 13 relevant housing associations were identified. In addition, a search of the legal form Kooperatinė bendrovė was conducted in the official business register (LTU_2). This search revealed 3 potential candidates, however they were not included in the inventory due to lack of verification of whether activities target the energy sector. But there have recently been many projects linked to housing retrofitting with some installing renewable energy using solar panels for heating. An issue here is that it is not clear whether these renovation projects are initiated by citizens living in the apartments, or whether the renovation is initiated by the municipality or private entrepreneurs and developers. To summarize, we did not include any retrofitting project that did not disclose information that they included PV-systems or other heating systems.

Validation & completeness

Data accuracy: Cross-checking of information on websites, documents publicly available and discussion with a group of experts from Lithuania (Banioniene *et al.*). **Data collection errors:** Four eyes principle, automated check for duplicates and wrong data formats during generation of data implementation files. **Validation of aggregates/Representativeness of sample:** The probability is high that additional housing associations exist, but are not registered. Our data therefore provide a conservative estimate. The topic of community energy is only emerging and citizens face high financial, social, and organizational barriers as confirmed by the group of experts. We concluded the discussion that the initial collection of a limited number of housing associations is justified. Also, cooperative forms are still confronted with historical prejudices, originating from negative experiences during the socialist era (Ruggiero *et al*. 2019). The authors do not report any existing initiative. The implementation of EU Directives is ongoing (European Commission 2020).

Contributors

Valeria Jana Schwanitz, Ingrid Knutsdotter Koren, Constantin von Beck

References

LTU_1_DB Database of community energy projects in the BSR,
http://old.lei.lt/co2mmunity/?s=lithuania&category&location&a=true&paged=1

Other References

European Commission. *Commission staff working document, Assessment of the final national energy and climate plan of Lithuania*. SWD (2020) 914 final. https://ec.europa.eu/energy/sites/default/files/documents/staff_working_document_assessment_necp_lithuania_en.pdf (2020).

Justina Banioniene, Elena Vitkauskaite, Lina Dagiliene, Manuel Morales; University of Kaunas, personal communication, January 11, 2022.

Ruggiero, S., Isakovic, A., Busch, H., Auvinen, K. & Faller, F. *Developing a joint perspective on community energy: Best practices and challenges in the Baltic Sea region*. Co2mmunity project, Working paper No. 2.3. http://co2mmunity.eu/wp-content/uploads/2019/03/Co2mmunity-working-paper-2.3.pdf (2019).

Luxembourg (LUX)

Methodological approach

Cooperatives were identified through filtering entities listed in the "Yellow pages" of Luxembourg (LUX_1_DB) for relevant business activities based on the internal classification system of the database and by checking relevant entries from LUX_1_DB in the official business register (LUX_2_OR) for relevant legal forms (*Société civile* (civil company), *Société coopérative* (cooperative society), *Association sans but lucratif* (non-profit association), Société coopérative organisée sous forme de société anonyme). Basic administrative data was gathered from LUX_1_DB and LUX_2_OR. This includes name, national identifier, address, year of foundation, capital, NACE classification, number of employees and legal form. NACE classification letter codes refer to the legal section, where B (cooperatives) are a commercial company, E are Civil Societies, and F are Non-Profit Associations. Data on associated energy production units was collected from individual websites (LUX_3_O), if available. Size of membership shares was collected from statutes that were generally available on the initiatives' individual websites.

Activity categories used for filtering in LUX_1_DB *(Bold faced categories represent the categories with the most hits)*: Distribution of electricity; Gas distribution; Distribution and supply of electricity; **Electricity Generation**; Gas production; Electricity transport; Gaz transport; Power distributor; Medium voltage distribution; Energy project engineering; Heating; Electricity, lighting; Lighting equipment - Public; **Alternative energy**; Energy; Energy audit; Carbon footprint; Carbon neutrality; Env. Consultancy; Sustainable development; Production, transport and distribution of biomass pellets; Production, transport and distribution of wood pellets. Trading - Wood pellets; Carsharing; Electric vehicle rental; Electric Motorbike; Electric scooter; Electric bike; Hybrid bicycle and electric bike.

Validation & completeness

Data accuracy: cross-checking of basic administrative data from LUX_1_DB and LUX_2_OR. If available, cross checking with individual websites (LUX_3_O). **Data collection errors:** Four eyes principle, automated check for duplicates and wrong data formats during generation of data implementation files. **Validation of aggregates/Representativeness of sample:** According to the Bridge Report (2019), 'a couple' of cooperatives engaging in the energy transition exist. We are able to identify 66 initiatives (44 citizen-led initiatives and 22 energy communities with limited citizen-leadership).

Contributors

Valeria Jana Schwanitz, Timothy Marcroft, Philippa Roots, Constantin von Beck

References

LUX_1_DB, Editus, "Yellow pages", https://www.editus.lu/en

LUX_2_OR, Luxembourg business register, https://www.lbr.lu

LUX_3_O, Initiatives' websites, (social) media, n/a

Additional references

Bridge Project. *Energy Communities in the EU - Task Force Energy Communities*. Bridge project, Report D3.12.d. https://www.h2020-bridge.eu/wp-content/uploads/2020/01/D3.12.d_BRIDGE_Energy-Communities-in-the-EU-2.pdf (2019).

Malta (MLT)

Methodological approach

The identification of initiatives was conducted through manual checking of lists of cooperatives provided by the databases Koperattivi Malta (MLT_1_DB) and Malta Co-operative Federation (MLT_2_DB), and further through Google searches. Only one potential candidate was identified (Enerġija Posittiva), but additional information was very limited (including whether or not this initiative still exists). Another candidate is Malta's first communal PV farm, where individual citizens could join through a bidding competition. However, it is not clear whether the criteria for democratic decision making holds, which is why we include the candidate but do not tag it additionally as a citizen-led project. For this initiative, activity data was available on the website of the Malta Energy and Water Agency (MLT_3_O).

Validation & completeness

Data accuracy: Limited information only, unclear if one of the initiatives still exists. No entry in the database. **Data collection errors:** Four eyes principle, automated check for duplicates and wrong data formats during generation of data implementation files. **Validation of aggregates/Representativeness of the search:** Apart from the three mentioned sources no further source of information or previous study was identified. We are confident to have fully covered the current state of development well, also considering the review of the current state of implementation of the EU directives promoting energy communities/self-consumption (European Commission 2020). Because of its size, the Maltese electricity production is centralized under the governance of just one company. Although cooperative law exists, the financial barriers in addition to the technical/organizational barriers are high. The concept of community energy as well as similar forms have not formally been established yet in Malta.

Contributors

Constantin von Beck, Ingrid Knutsdotter Koren

References

MLT_1_DB, Koperattivi Malta, https://cooperatives-malta.coop/

MLT_2_DB, Malta Co-operative Federation, https://maltacooperativefederation.coop/

MLT_3_O, The Energy and Water Agency, https://www.energywateragency.gov.mt/other-energy-projects/

Additional references

European Commission. *Commission staff working document, Assessment of the final national energy and climate plan of Malta*. SWD (2020) 917 final. https://ec.europa.eu/energy/sites/default/files/documents/staff_working_document_assessment_necp_malta_en.pdf (2020).

Netherlands (NLD)

Methodological approach

Initiatives were identified through a search via service operators Drimble and Bedrijvenmonitor to the business register KVK for the legal forms *Coöperatie* (cooperative), *Vereniging* (association, society), *Stichting* (foundation, trust) listed under energy-related Dutch industrial sector codes or including relevant keywords in the entity name. Additional initiatives were extracted through a list of citizen energy projects provided by HierOpgewekt (NLD_1_R - NLD_3_DB) and from lists of citizen energy projects provided by NLD_5_DB, NLD_6_DB and NLD_7_DB. Administrative and financial data was gathered from Dutch business registers (NLD_4_DB and NLD_10_DB) and initiatives' websites (NLD_11_O). Information on energy generation units was gathered from NLD_1_R - NLD_3_DB, NLD_4_DB - NLD_7_DB, and NLD_11_O - NLD_13_O. Keywords used for the search included: energiek, zonne, fotovoltaïsch, varmte, water, waterkracht, hydro, wind, biomassa, geothermie, hernieuwbar, licht, duurzaam; the following NACE codes were used to analyze activities: 35, including subcategories.

Validation & completeness

Data accuracy: cross-referencing of data from various NGOs (NLD_1_R - NLD_3_DB, NLD_5_DB - NLD_7_DB) with websites (NLD_11_O), business registers (NLD_4_DB and NLD_10_DB) and open access map applications (NLD_12_O, NLD_13_O). **Data collection errors:** Four eyes principle, automated check for duplicates and wrong data formats during generation of data implementation files. **Validation of aggregates/Representativeness of sample:** Comparison with previous studies. Comparison of initiatives gathered through our systematic search of business registers and the dataset provided by HierOpgewekt (NLD_1_R - NLD_3_DB). Comparison of our data on total investments/assets with anonymized open data provided by Dutch Chamber of Commerce (NLD_8_OR, NLD_9_OR). Hieropgewekt 2020 (NLD_1_R) lists for 2019 a total of 582 initiatives with about 8500 members, wind capacity of 193 MW, PV capacity of 119 MWp, as well as 54 district heating projects. Hieropgewekt 2021 (NLD_2_R) reports for 2020, 623 initiatives with 97000 members, 229.9 MW of wind capacity, 166.4 MWp of PV and 77 district heating projects. Hufen 2015 counted in 2015 16 wind cooperatives and 91 non-wind cooperatives, Boon 2014 for 1980, 10 initiatives and in 2012 a total of 60.

Contributors

August Wierling, Jan Pedro Zeiss, Constantin von Beck, Heather Arghandeh Paudler, Negar Safara Nosar

References

NLD_1_R, Hier Opgewekt (2020), Lokale Energie Monitor 2019, https://www.hieropgewekt.nl/lokale-energie-monitor

NLD_2_R, Hier Opgewekt (2021), Lokale Energie Monitor 2020, https://www.hieropgewekt.nl/lokale-energie-monitor

NLD_3_DB, Hier Opgewekt (2021), Lokale Energie Monitor 2020, Excel dataset, https://www.hieropgewekt.nl/lokale-energie-monitor

NLD_4_DB, Bedrijvenmonitor, Dutch Business Register, https://bedrijvenmonitor.info/

NLD_5_DB, Energie Sammen, https://energiesamen.nu/pagina/1/jouw-belangenvereniging/35/leden-en-supporters

NLD_6_DB, Energie Van Ons, https://energie.vanons.org/bij-jou-in-de-buurt/

NLD_7_DB, Zon op Nederland, https://www.zonopnederland.nl/projecten-in-productie/

NLD_8_OR, KVK Handelsregister Open Data Set, https://www.kvk.nl/producten-bestellen/koppeling-handelsregister/kvk-handelsregister-open-data-set/

NLD_9_OR, KVK Jaarrekeningen Open Data Set, https://www.kvk.nl/producten-bestellen/koppeling-handelsregister/kvk-jaarrekeningen-open-data-set/

NLD_10_DB, Drimble - business register, https://drimble.nl/

NLD_11_O, Initiatives' websites, (social) media, n/a

NLD_12_O, Windturbines - ArcGIS map containing information on wind turbines, https://www.arcgis.com/home/item.html?id=4d2e288f6d26406aadba730414faf7a0

NLD_13_O, Open Infrastructure Map - Open Street Map application containing information on energy infrastructure, https://openinframap.org

Additional references

Boon, F. & Dieperink, C. Local civil society based renewable energy organizations in the Netherlands: Exploring the factors that stimulate their emergence and development. *Energy policy*. 69, 297–307; https://doi.org/10.1016/J.ENPOL.2014.01.046 (2014).

Hufen, J. & Koppenjan, J. Local renewable energy cooperatives: revolution in disguise? *Energy, sustainability and society*. 5/1; https://doi.org/10.1186/s13705-015-0046-8 (2015).

Norway (NOR)

Methodological approach

Information is only available from a study on the Norwegian community energy sector conducted by the Norwegian Water Resources and Energy Directorate in 2018 (NOR_1_R). This report lists general information only and concludes that detailed information is restricted due to data legislation and privacy. 5 initiatives were reported to be active, with an additional 25 in implementation stages. Further details are not available. The database provides the link to the report and counts the initiatives and projects.

Validation & completeness

Not applicable.

Contributors

Ingrid Knutsdotter Koren, Jan Pedro Zeiss, August Wierling

References

NOR_1_R, NVE report,
https://thema.no/wp-content/uploads/THEMA-Reort-2018-20-Local-Energy-Communities-Report-Final.pdf

Poland (POL)

Methodological approach

Legislation that allows for the foundation of energy communities is under construction. As Poland does not have an official database, information was sourced from governmental, institutional, and company websites, reports, and literature. Only one cooperative exists, but no associated activities, and one is under application as of July 2021 according to POL_1_O. Furthermore, the government opts for so-called "energy clusters" (POL_2_O). The third main legal form is housing cooperatives. Information about renewable installations owned and/or managed by initiatives was collected from various distributed sources as no open-access administrative database for micro RES installations exists.

Validation & completeness

Data accuracy: Cross-referencing of data from different data sources i.e. POL_1_O, POL_2_O Internet search, telephone follow-ups, internet online query, data from the COMETS project: Polish National Research Team workshops, Interviews (IDI), web survey (CAWI). **Data collection errors:** Four eyes principle and several rounds of discussions with all contributing authors, two of which are experts on the topic in Poland, automated check for duplicates and wrong data formats during generation of data implementation files. **Validation of aggregates/Representativeness of sample:** We are confident that we capture the current development in the country. KOWR 2021 lists 1-2 initiatives but without projects: 66 certified pilot clusters are operating as of 07/2021 (Ministerstwo Aktywów Państwowych 2021). They are contributing about 150 MW under the assumption of full ownership and based on an average of

announced capacity by 10 pilot clusters of up to 2.5 MW. 45 housing cooperatives and associations invested in RES (PV). Assuming the average apartment/ house size has installations of 10-40 kW, the estimate is about 0.045 to 1.8 MW as of spring 2021. Note that many organizations only started operating after 2018 and reporting on installations is anecdotal.

Contributors

Tadeusz Józef Rudek, Valeria Jana Schwanitz, Wit Hubert, August Wierling

References

POL_1_O, KOWR 2021, The National Support Centre for Agriculture (Krajowy Ośrodek Wsparcia Rolnictwa), https://www.kowr.gov.pl/uploads/pliki/DI/Spóldzielnie%20enrgetyczne/Rejestr%20spółdzielni%20energetycznych/Wykaz%20spółdzielni%20energetycznych_2021.05.11.pdf (Note: link broken)

POL_2_O, Ministerial registry, https://www.gov.pl/web/klimat/polityka-energetyczna-pol

POL_3_P, Jazinski *et al*., 2021, https://doi.org/10.3390/en14020319

Additional references

Ministerstwo Aktywów Państwowych (Ministry of State Assets). *Krajowy plan na rzecz energii i klimatu na lata 2021–2030 (National plan for energy and climate for 2021–2030)* (2019).

Portugal (PRT)

Methodological approach

Data were sourced from official registries, a non-official database, and cooperative's websites. In case of double referencing of data, the sources of information were used for cross-checking and completion of entries. No initiatives were found in the official business register (PRT_5_OR). Most of the initiatives (cooperatives registered for electricity distribution) were listed on the website of the Directorate for Energy and Geology (PRT_6_OR). In the cooperative's registry of the António Sérgio Cooperative for the Social Economy (CASES, PRT_1_OR), initiatives' entries were cross-checked and completed. Furthermore, a websearch for cooperatives' names yielded additional administrative, financial, demographic, and activity data from their websites (PRT_4_O). Data on energy generation units could be gathered only from the website of the country-wide initiative Coopernico (PRT_3_DB), and validated by direct communication with the cooperative. Search terms included: energia, coop, cooperativa, CRL, União de Cooperativas, Federação de Cooperativas, and Confederação de Cooperativas.

Validation & completeness

Data accuracy: Datasource CASES (PRT_1_OR) were cross-referenced with DGEG (PRT_6_OR) and Portugal Energia (PRT_2_O). **Data collection errors:** Consistency-check by four eyes principle, automated check for duplicates and wrong data formats during generation of data implementation files. **Validation of aggregates/Representativeness of sample:** Comparison with previous studies. **Further remarks:** PRT_6_OR has rebuilt its website at the end of 2021. The list of electricity distribution cooperatives is no longer accessible. A similar list (likely copied

from PRT_6_OR) is available on Portugal Energia (PRT_2_O), and updated data on cooperatives are also available at CASES (PRT_1_OR).

Contributors

Tobias Kraudzun, Valeria Jana Schwanitz, Nico Primo

References

PRT_1_OR, CASES. António Sérgio Cooperative for the Social Economy, https://credencial.cases.pt/pt-PT/0/PCR/Arqui/PCR_Menu_COOPERATIVASCREDENCIADAS?nav=L5G9U2cC

PRT_2_O, Portugal Energia (Awareness building for energy transition, EU-funded), https://www.portugalenergia.pt/agentes/

PRT_3_DB, Coopernico Coop production units, accessed 01.10.2021, https://www.coopernico.org/pt/projects

PRT_4_O, Initiatives' websites, (social) media, n/a

PRT_5_OR, ePortugal: Empresa Online, https://eportugal.gov.pt/empresas/Services/Online/Pedidos.aspx?service=PNS

PRT_6_OR, Direção Geral de Energia e Geologia (DGEG), https://www.dgeg.gov.pt/pt/areas-setoriais/energia/energia-eletrica/servicos-e-redes/comercializadores-de-eletricidade-de-ultimo-recurso/ (website now reorganized)

Romania (ROU)

Methodological approach

Search for cooperatives began with the analysis of literature. Cebotari 2019 collected data between 2015-2016, pointing to two community-owned renewable energy initiatives. However, as discussed in the paper, the community benefit in both is assessed to be small and decision-making lies with the local government which is why we did not include the candidates in the inventory. A recently founded initiative was identified through internet research (ROU_1_O). The initiative was verified through ROU_2_DB.

Validation & completeness.

Data accuracy: Comparison of self-reported data obtained from the website with information sourced from official registries. **Data collection errors:** Consistency-check by four eyes principle. **Validation of aggregates/Representativeness of sample:** We are likely covering the current activities in Romania (RESCOOP 2021).

Contributors

Simon Dufner, Ingrid Knutsdotter Koren, Constantin von Beck

References

ROU_1_O, Cooperativa de Energie, https://cooperativadeenergie.ro/despre-ce/

ROU_2_DB, firme.info, COOPERATIVADEENERGIE.RO SOCIETATE COOPERATIVA EUROPEANA CU RASPUNDERE LIMITATA - CUI 41659859 firme.info

Additional references

Cebotari, S. Against all odds: Community-owned renewable energy projects in North-West Romania. *ACME: An International journal for critical geographies*, 18/2, 513-528; https://acme-journal.org/index.php/acme/article/view/1556 (2019).

Rescoop. News posted in July 2021. https://www.rescoop.eu/news-and-events/news/july-success-story-the-beginning-of-community-energy-in-romania (2021).

Slovakia (SVK)

Methodological approach

Identification of initiatives began with a search in Atlas of the use of renewable energy sources in Slovakia (SVK_1_R) for energy generation units with owner names including the term "Družstvo" ("cooperative"). The extraction of administrative data for generation unit owners identified in the initial search came from the business register, SVK_2_OR, and a third party data provider (SVK_3_O). The business register (SVK_2_OR) was searched for key terms, filtered for legal form "Družstvo", to check for energy-related statements of purposes for the identified entities. A further Google search was conducted for "energie/elektriny + Družstvo". Search terms included the following: en, ener, fotovoltaické elektriny, EKO, green.

Validation & completeness

Data accuracy: Data is based on SVK_1_R published in 2012. Inactive initiatives were marked as such. Searching for further initiatives in SVK_2_OR and Google has only yielded three more results. **Data collection errors:** Consistency-check by four eyes principle, automated check for duplicates and wrong data formats during generation of data implementation files. **Validation of aggregates/Representativeness of sample:** n/a. **Further remarks:** The vast majority of initiatives are agricultural cooperatives engaging in biogas or cooperatives active as consultants for energy installations. There is little indication of initiatives being citizen-led. Few initiatives operate heating plants, based on e.g. woodchips. Some initiatives operate PV plants. Only one initiative with renewable energy as their primary objective was identified.

Contributors

Lukas Müller, Yann Robio du Pont

References

SVK_1_R, Atlas of the use of renewable energy sources in Slovakia, https://sclib.svkk.sk/sck01/Record/000417744/Details#description

SVK_2_OR, Slovakian business register, https://www.orsr.sk

SVK_3_O, information provider, https://finstat.sk/information-of-slovak-companies

Slovenia (SVN)

Methodological approach

Primary identification of initiatives was conducted through the Slovenian business register (SVN_1_OR) by filtering for the following legal forms (Agriculture coop, Cooperative, Housing coop, Limited liability coop (z.o.o.), Local communities, No-liability coop (z.b.o.), Other collectives, Privately owned coop) and energy related industrial sector codes (35.111, 35.112, 35.119, 35.120, 35.130, 35.140). We also conducted a search in the Slovenian register of energy generation units (SVN_2_OR) for unit owner names containing the legal form abbreviations z.o.o and z.b.o. One additional project/initiative was found on the website of the COMPILE project (SVN_3_O). Administrative data was gathered from SVN_1_OR and data on energy generation units come from SVN_2_OR. Main activities were analyzed using national economic activity codes: 35.111 Production of electricity in HE generations facilities, 35.112 Production of electricity in thermal power stations 35.119, Other production of electricity 35.120, Transmission of electricity 35.130, Distribution of electricity 35.140, Trade of electricity. Legal forms searched: Agriculture coop, Cooperative Housing coop, Limited liability coop (z.o.o.), Local communities, No-liability coop (z.b.o.), Other collectives, Privately owned coop, etc.

Validation & completeness

Data accuracy: n/a. **Data collection errors:** Four eyes principle, automated check for duplicates and wrong data formats during generation of data implementation files. **Validation of aggregates/Representativeness of sample:** Comparison with previous studies. Cross-referencing of initiatives identified in the various sources. When compared with previous studies we find that we are likely to capture existing initiatives. According to Palm 2020, citizen-led energy initiatives are still in their infancy in Slovenia. Palm 2020 identified 2 existing initiatives, and the Compile project 2019 identified 1 additional initiative. In our inventory, we report on 8 initiatives, additionally sourcing from SVN_1 and SVN_2, which identified 5 additional initiatives with relevant legal forms and activities.

Contributors

Ingrid Knutsdotter Koren, Valeria Jana Schwanitz

References

SVN_1_OR, Slovenia business register, https://www.ajpes.si/prs/default.asp

SVN_2_OR, Slovenia Energy register list of units, https://poi.borzen.si/register/DevicesList.aspx

SVN_3_O, COMPILE Project website, https://www.compile-project.eu/news/installation-and-connection-of-pv-in-luce-slovenia/

Additional references

Compile Project. *Installation and connection of PV in Luče, Slovenia*. https://www.compile-project.eu/news/installation-and-connection-of-pv-in-luce-slovenia/ (2019).

Palm, J. *et al*. *Deliverable D3, Description of polycentric settings in the partner countries*. New Clean Energy Communities in a Changing European Energy System (NEWCOMERS) project, Deliverable D3. https://www.researchgate.net/publication/342336463_New_Clean_Energy_Communities_in_a_Changing_European_Energy_System_NEWCOMERS_Deliverable_D3_1_Description_of_polycentric_settings_in_the_partner_countries/link/5f16cb30a6fdcc9626a437e1/download (2020).

Spain (ESP)

Methodological approach

The search started with a list of 12 initiatives published in Heras-Saizarbitoria *et al.* 2018. This list was extended and complemented with administrative information sourced from ESP_1_OR and ESP_2_O. As the information on members, activities, production units, and finances are not officially registered and rely on individual internet searches, the coverage is partially incomplete, in particular for production units, finances, and number of members, customers. Internet searches also occasionally led to the identification of additional initiatives. The two bigger initiatives (Somenergia, Enercoop) provide access to their annual reports which allows us to cover them rather completely.

Validation & completeness

Data accuracy: Data is based on ESP_1_OR and ESP_2_O. **Data collection errors:** Consistency checked by four eyes principle, cross checks between different data sources, and an automated check for duplicates and formatting during data implementation. **Validation of aggregates/Representativeness of sample:** Compared with the literature, we are confident that we cover about 80% of the initiatives as of the end of 2021.

Contributors

Tobias Kraudzun, August Wierling, Jan Pedro Zeiss, Heather Arghandeh Paudler, Constantin von Beck, Ingrid Knutsdotter Koren, Simon Dufner, Valeria Jana Schwanitz, Tim Marcroft

References

ESP_1_OR, Registry of OMIE - Spanish Electricity Market Operator, Historical list of codes assigned to agents that have participated in the power market, https://www.omie.es/en/list-agents

ESP_2_O, Union of Renewable Energy Producers, https://www.unionrenovables.coop/

Additional references

Heras-Saizarbitoria, I., Sáez, L., Allur, E. & Morandeira, J. The emergence of renewable energy cooperatives in Spain: A review. *Renewable and Sustainable Energy Reviews.* 94, 1036–1043; https://doi.org//10.1016/j.rser.2018.06.049 (2018).

Sweden (SWE)

Methodological approach

The identification of initiatives was based on previous studies (SWE_6_P and SWE_8_P) and a search in the Swedish business register (SWE_3_OR) for relevant legal forms (see Tab. 1, main document) and business activities. For wind turbines with owners classified under category "S" (i.e., cooperatives, associations, foundations), a search was conducted in Vindstat (SWE_4_P). Then information was extracted through a search in Vindbrukskollen (SWE_1_DB) for wind turbines with turbine owner names including the term "förening". Administrative and financial data were gathered from SWE_2_OR, SWE_3_OR, SWE_6_P - SWE_11_O. Data on energy generation units were collected from SWE_1_DB, SWE_4_P, SWE_5_DB, SWE_6_P and SWE_11_O. Data to analyze activities was based on Swedish industrial sector codes (SNI-codes): 3511, 3512, 3513, 3514, 3521, 3522, 3523, 353, 3821, 4612, 4571, 49311, 49319, 64999, 71123, 71124, 77110, 71129.

Validation & completeness

Data accuracy: cross-referencing of data from different data sources. **Data collection errors:** Four eyes principle, automated check for duplicates and wrong data formats during generation of data implementation files. **Validation of aggregates/Representativeness of sample:** Comparison with previous studies. Cross-referencing of initiatives identified from the various sources. We report over 300 initiatives, a significantly higher number compared to aggregates from other papers. A likely reason for this is that our database includes liquidated and deleted initiatives, housing cooperatives, and initiatives under many legal forms relevant for our scope. Many of the deleted initiatives were identified in a book by Wizelius 2010, which lists 72 initiatives. Magnusson 2018 lists a total number of 78 wind cooperatives, Magnusson 2019 reports a total number of 140 citizen-led energy projects (32 of them are ecovillages) and Hewitt 2019 points to a total number of 112 initiatives (25 of these are ecovillages). **Further remarks:** Standardized data sources on energy generation units are only available for wind turbines (SWE_1 and SWE_4). Data collection for other energy generation technologies relied on information from initiatives' websites.

Contributors

Ingrid Knutsdotter Koren, Jan Pedro Zeiss, August Wierling

References

SWE_1_DB, VINDBRUKSKOLLEN, Swedish Wind Turbine register, https://vbk.lansstyrelsen.se/en

SWE_2_OR, BOLAGSVERKET, Swedish business register, https://snr4.bolagsverket.se/snrgate/startIn.do

SWE_3_OR, ALLABOLAG (provides business information; allows search by industrial sector), https://www.allabolag.se

SWE_4_P, VINDSTAT, Yearly and monthly reports on power production of Swedish wind turbines, http://vindstat.com/rapporter-2/

SWE_5_DB, THE WIND POWER, Global Wind farms database, https://www.thewindpower.net/store_windfarms_view_all_en.php

SWE_6_P, Wizelius, T., Vindkraft tillsammans: handbok för vindkooperativ, 1st ed., Stockholm, Vindform Förlag, 2010.

SWE_7_DB, RATSIT, Search engine for businesses and private persons in Sweden, https://www.ratsit.se/

SWE_8_P, Hewitt *et al*. (2019), https://doi.org/10.1016/j.rser.2019.109489

SWE_9_DB, Allabrf - register of housing cooperatives, https://www.allabrf.se/

SWE_10_DB, Merinfo - online business register, https://www.merinfo.se

SWE_11_O, Initiatives' websites, (social) media, n/a

SWE_12_P, Magnusson 2018 - ecovillages, https://www.tandfonline.com/doi/pdf/10.1080/14702541.2018.1465199?needAccess=true

Additional references

Magnusson, D. Going back to the roots: the fourth generation of Swedish eco-villages. *Scottish geographical journal*. 134/3-4, 122-140; https://doi.org/10.1080/14702541.2018.1465199 (2018).

Magnusson, D. & Palm, J. Come Together—The Development of Swedish Energy Communities. *Sustainability*. 11/4, 1056; https://doi.org/10.3390/su11041056 (2019).

Switzerland (CHE)

Methodological approach

Identification of initiatives took place through Zefix (CHE_1_OR), the official business register, utilizing keywords based on CHE_2_P, extended by our own keywords. The identified initiatives were then searched via Google. All additional information (e.g. installed capacity, fields of activities, financial numbers, statutes) was taken from the individual webpages (CHE_3_O), if available. If no homepage was available, articles in newspapers, or information from third parties (e.g., government, municipality, suppliers, partners) were incorporated. Additionally, e-mails were sent to initiatives to ask for further information (CHE_4_O).

Keywords used to identify initiatives in CHE_1_OR included: Elektra (used for historic cooperatives), Energie, energia, énergie, Solar, solare, Solaire, Sonne, Sunne, photovoltaik, photovoltaïque, fotovoltaico, Wärme, Heiz, Chauffage, riscaldamento, chaleur, termico, caldo, calore, Strom, Elektrizität, éléctricité, elettrica, elettrici, électrique, Wind, éolienne, vent, Wasser, eau, aqua, hydro, Biomasse, biomasse, biomassa, Holzschnitzel, trucili di legno, copeaux de bois, Geothermie, géothermie, thermo, energia geotermica, erneuerbar, renouvelable, rinnovabile, eco, Autovoltaic (Selbstbau/DIY-coops), Kraftwerk (power plant), Licht (light contracting), Zukunft (transition activities/future planning).

Validation & completeness

Data accuracy: cross-checking of information on websites, documents publicly available, media articles, and directly approaching founders, CEOs, employees, or members of initiatives via mail.

Data collection errors: Four eyes principle, automated check for duplicates and wrong data formats during generation of data implementation files. **Validation of aggregates/Representativeness of sample:** In comparison with previous studies, the number of initiatives seems reasonable. Some initiatives with names other than the utilized keywords may be neglected. Some types of initiatives were explicitly excluded by Rivas *et al*. (2018) but are included in our sample, e.g., initiatives engaged in selling biomass fuels, woodchips, etc. Hence, the total number of initiatives may differ compared to Rivas *et al.* (2018). However, Rivas *et al*. (2018) report a total of 289 initiatives, 86 of which generate electricity from PV. Given the declining trend in grid operating initiatives, our total number of initiatives appears reasonable. **Further remarks:** Three large initiatives were identified accounting for the majority of installed capacity and members. The reported figures thus reflect the general situation in Switzerland well. Detailed information on individual production units is available for 70% of all initiatives that reported their total installed capacity. The number increases to 95% when the largest initiative is excluded. However, information on initiatives engaging in heat is scarce, and thus not reliable.

Contributors

Lukas Müller, August Wierling

References

CHE_1_OR, Zefix Handelsregister, www.zefix.ch

CHE_3_O, Initiatives' websites, (social) media, n/a

CHE_4_O, email survey by Lukas Müller

Additional references

Rivas, J., Schmid, B. & Seidl, I. Energiegenossenschaften in der Schweiz: Ergebnisse einer Befragung. *WSL Berichte*. 71, 1-108; https://www.wsl.ch/de/publikationen/energiegenossenschaften-in-der-schweiz-ergebnisse-einer-befragung.html (2018).

3 Guidelines to access the database

The "Energy by the people (ENBP)" database is accessible from the inventory dataverse.no. All data is contained in a single Turtle file (2022_ENBP_inventory.ttl). This file can be analyzed in various ways. The most basic approach is to upload it in an editor and search for corresponding keywords. A more sophisticated possibility is to load the turtle file into a triple-store-solution, e.g., Apache Jena Fuseki (https://jena.apache.org/documentation/fuseki2/), and to explore the database with the help of SPARQL commands. The latter also allows downloading selected information into .csv files. Below we provide the following: 1) a brief explanation of the TTL triples and SPARQL query language, 2) a number of useful SPARQL commands for database queries, and 3) extended PREFIX and variable lists.

TTL triples and SPARQL query language

The Turtle language consists of the triple "subject-predicate-object". For example, *?cai schema:name ?cainame* contains the subject (?cai), predicate (schema:name), and object

(?cainame). In other words, this command asks to find all subjects (?cai) and objects (?cainame) with the schema:name predicate. The comprehensive SPARQL query for such a search looks like:

```
PREFIX dc: <http://purl.org/dc/elements/1.1/>
PREFIX enbp: <http://www.your-domain/>
PREFIX wikidata: <https://www.wikidata.org/entity/>
PREFIX schema: <http://schema.org/>

SELECT ?cainame

WHERE {
    ?cai schema:name ?cainame.
}
```

The SPARQL query language allows screening of these triples, returning all triples which follow a particular pattern. SPARQL offers several commands to search and manipulate data. The most often used command is the combination of SELECT and WHERE. After the SELECT statement, the item to be returned is stated. In the aforementioned example, the item to be returned,

```
SELECT ?cainame
```

is the name of all CAIs in the database. In the WHERE clause,

```
WHERE {
    ?cai schema:name ?cainame.
}
```

the pattern that is to be compared with is given.

Note that the most basic triple entry is the line where subject, predicate, and object are separated by spaces and the statement ends with a dot (period). An extended list of variables is provided at the end of this section. The prefixes listed above the SELECT and WHERE command combination are prefix declarations for abbreviating URLs. An extended list of prefixes is also included at the end of this section.

Another useful query may be to find all CAIs in a specific country. For example, a query to find the names of all initiatives in Slovenia, the comprehensive SPARQL query for the search looks like

```
PREFIX dc: <http://purl.org/dc/elements/1.1/>
PREFIX enbp: <http://www.your-domain/>
PREFIX wikidata: <https://www.wikidata.org/entity/>
PREFIX schema: <http://schema.org/>

SELECT ?cainame

WHERE {
    ?cai a enbp:CAI.
    ?cai wikidata:P298 "SVN".
    ?cai schema:name ?cainame.
}
```

Explanation: The three statements in the WHERE clause select

- all CAIs (?cai a enbp:CAI),

- which are from Slovenia (?cai wikidata:P298 "SVN"), and
- select patterns with the predicate (?cai schema:name ?cainame) and store matching object entries into the variable ?cainame.

The variable after the SELECT statement is the one that will be returned if the command is run.

Make sure that the same subject (?cai) is given in all three statements so that triples are selected for the same initiative. As in this case of turtle statements, several statements with the same subject can be replaced by another syntax to increase readability. This is done by combining them into one ending with a single ".", where the different parts are separated by a semicolon ";". This shortens the query considerably to just:

```
PREFIX dc: <http://purl.org/dc/elements/1.1/>
PREFIX enbp: <http://www.your-domain/>
PREFIX wikidata: <https://www.wikidata.org/entity/>
PREFIX schema: <http://schema.org/>

SELECT ?cainame ?city

WHERE {
    ?cai a enbp:CAI; wikidata:P298 "SVN"; schema:name ?cainame.
}
```

The example above can be expanded to include the city locations of all initiatives in Slovenia with the following SPARQL query:

```
PREFIX dc: <http://purl.org/dc/elements/1.1/>
PREFIX enbp: <http://www.your-domain/>
PREFIX wikidata: <https://www.wikidata.org/entity/>
PREFIX schema: <http://schema.org/>

SELECT ?cainame ?city

WHERE {
    ?cai a enbp:CAI.
    ?cai wikidata:P298 "SVN".
    ?cai schema:name ?cainame.
    ?cai schema:addressLocality ?city.
}
```

Explanation: The four statements in the WHERE clause select

- all CAIs (?cai a enbp:CAI),
- which are from Slovenia (?cai wikidata:P298 "SVN"), and
- select patterns with the predicate (?cai schema:name ?cainame) and stores matching object entries into the variable ?cainame, and
- select patterns with the predicate (?ca schema:addressLocality ?city) and store matching objects into the variable ?city.

The variables (*?cainame ?city*) after the SELECT statement are the ones returned when the command is run.

The shortened query is the following:

```
PREFIX dc: <http://purl.org/dc/elements/1.1/>
PREFIX enbp:  <http://www.your-domain/>
PREFIX wikidata: <https://www.wikidata.org/entity/>
PREFIX schema: <http://schema.org/>

SELECT ?cainame ?city

WHERE {
      ?cai a enbp:CAI; wikidata:P298 "SVN"; schema:name ?cainame; schema:addressLocality ?city.
}
```

A query that **lists the names of countries for all initiatives that are classified as CAI** (collective action initiatives) is:

```
PREFIX dc: <http://purl.org/dc/elements/1.1/>
PREFIX enbp:  <http://www.your-domain/>
PREFIX wikidata: <https://www.wikidata.org/entity/>
PREFIX schema: <http://schema.org/>

SELECT ?cainame ?country

WHERE {
  ?cai a enbp: CAI; schema:name ?cainame ; wikidata:P298 ?country.
}
```

A longer query which reports **name, status, street, city, postal code, and Legal Form Code** has the following form:

```
PREFIX dc: <http://purl.org/dc/elements/1.1/>
PREFIX enbp:  <http://www.your-domain/>
PREFIX wikidata: <https://www.wikidata.org/entity/>
PREFIX schema: <http://schema.org/>
PREFIX gleif-L1: <https://www.gleif.org/ontology/L1/>

SELECT ?cainame ?street ?city ?postal ?status ?gleif

WHERE {
      ?cai a enbp: CAI.
      ?cai wikidata:P298 "SVN".
      ?cai schema:name ?cainame.
      ?cai schema:streetAddress ?street.
      ?cai schema:addressLocality ?city.
      ?cai schema:postalCode ?postal.
      ?cai enbp: status ?status.
  OPTIONAL { ?cai gleif-L1:hasLegalForm ?gleif}
}
```

Note that in the PREFIX list above the SELECT and WHERE command combination, the prefix for GLEIF is added. The prefix addition is necessary since the pattern in the OPTIONAL keyword includes a query for the Entity Legal Form (GLEIF) Codes. The OPTIONAL keyword can be used for entries, which are not necessarily assigned, so that the output leaves this information blank, but still returns all remaining information. If no OPTIONAL statement is used, only results with

complete information are returned. In the above example, results will be returned even if a specific CAI does not have a GLEIF code.

Useful SPARQL commands for database queries, country-level

Example: **Return all information available for just one country.** Run the script below, replacing the three-letter country code with the desired country. Here, Hungary - HUN - is used.

```
PREFIX dc: <http://purl.org/dc/elements/1.1/>
PREFIX enbp:  <http://www.your-domain/>
PREFIX wikidata: <https://www.wikidata.org/entity/>
PREFIX schema: <http://schema.org/>
PREFIX gleif-L1: <https://www.gleif.org/ontology/L1/>

SELECT ?s ?p ?o

WHERE {
    ?s wikidata:P298 "HUN"; ?p ?o
}
```

Example: **Count of subjects in the database for a country.** Here, Latvia - LVA - is used.

```
PREFIX dc: <http://purl.org/dc/elements/1.1/>
PREFIX enbp:  <http://www.your-domain/>
PREFIX wikidata: <https://www.wikidata.org/entity/>
PREFIX schema: <http://schema.org/>
PREFIX gleif-L1: <https://www.gleif.org/ontology/L1/>

SELECT DISTINCT ?s

WHERE {
    ?s wikidata:P298 "LVA"; ?p ?o
}
```

Example: **List an overview of important properties (Name, Street, City, Postal code, latitude, longitude, website, foundation year, dissolution year) for all initiatives in a given country.** Run the script below, replacing the desired country. Here, Austria - AUT - is selected.

```
PREFIX schema: <http://schema.org/>
PREFIX dbo: <http://dbpedia.org/ontology/>
PREFIX enbp: <http://www.your-domain/>
PREFIX wikidata: <https://www.wikidata.org/entity/>
PREFIX sc: <http://purl.org/science/owl/sciencecommons/>
PREFIX geo: <http://www.w3.org/2003/01/geo/wgs84_pos#>

SELECT ?cainame ?street ?city ?postal ?latitude ?longitude ?url ?start ?end

{
  ?cai a schema:Organization; wikidata:P298 "AUT"; schema:name ?cainame.
  OPTIONAL { ?cai schema:streetAddress ?street}
  OPTIONAL { ?cai schema:addressLocality ?city}
  OPTIONAL { ?cai schema:postalCode ?postal}
  OPTIONAL { ?cai geo:lat ?latitude}
  OPTIONAL { ?cai geo:long ?longitude}
```

```
  OPTIONAL { ?cai schema:url ?url}
  OPTIONAL { ?cai schema:foundingDate ?start}
  OPTIONAL { ?cai schema:dissolutionDate ?end}
}
```

Example: **List of types of production units for a given country.** To list all production unit types in a specific country, run the following script, replacing the country code with the desired country. Here, Slovakia - SVK - is selected.

```
PREFIX schema: <http://schema.org/>
PREFIX dbo: <http://dbpedia.org/ontology/>
PREFIX enbp: <http://www.your-domain/>
PREFIX wikidata: <https://www.wikidata.org/entity/>

SELECT ?unit ?unitname ?type

{
  ?unit a enbp:ProductionUnit; a ?type; enbp:hasName ?unitname; wikidata:P298 "SVK".
}
```

Example: **List all capacities of production units for a given country (except cogeneration plants).** Run the script below, adapting by changing the country code. Here, Italy - ITA - is selected.

```
PREFIX schema: <http://schema.org/>
PREFIX dbo: <http://dbpedia.org/ontology/>
PREFIX enbp: <http://www.your-domain/>
PREFIX wikidata: <https://www.wikidata.org/entity/>
PREFIX qudt-1-1: <http://qudt.org/1.1/schema/qudt#>
PREFIX gleif-L1: <https://www.gleif.org/ontology/L1/>
PREFIX qudt-1-1: <http://qudt.org/1.1/schema/qudt#>

SELECT ?unit ?unitname ?cap
WHERE
{
  ?unit a enbp:ProductionUnit; wikidata:P298 "ITA".
  OPTIONAL {?unit enbp:hasName ?unitname }
  OPTIONAL {?unit enbp:nameplateCapacity ?nameplate.  ?nameplate qudt-1-1:value ?cap}
}
```

Example: **List all cogeneration plant production units for a given country.** Run the script below, replacing the desired country. Here, Spain - ESP - is selected.

```
PREFIX schema: <http://schema.org/>
PREFIX dbo: <http://dbpedia.org/ontology/>
PREFIX enbp: <http://www.your-domain/>
PREFIX wikidata: <https://www.wikidata.org/entity/>
PREFIX qudt-1-1: <http://qudt.org/1.1/schema/qudt#>
PREFIX gleif-L1: <https://www.gleif.org/ontology/L1/>
PREFIX qudt-1-1: <http://qudt.org/1.1/schema/qudt#>

SELECT ?unit ?unitname ?cap_electric ?cap_thermal
WHERE
{
```

```
  ?unit a enbp:ProductionUnit; wikidata:P298 "ESP".
  OPTIONAL {?unit enbp:hasName ?unitname }
  OPTIONAL {?unit enbp:nameplateCapacityElectric ?nameplate1.  ?nameplate1 qudt-1-1:value ?cap_electric}
  OPTIONAL {?unit enbp:nameplateCapacityThermal ?nameplate2.  ?nameplate2 qudt-1-1:value ?cap_thermal}
}
```

Example: **List capacities along with ownership share.** To include ownership share of capacities in a chosen country, use the following statement. Here, Switzerland - CHE - is selected.

```
PREFIX schema: <http://schema.org/>
PREFIX dbo: <http://dbpedia.org/ontology/>
PREFIX enbp: <http://www.your-domain/>
PREFIX wikidata: <https://www.wikidata.org/entity/>
PREFIX qudt-1-1: <http://qudt.org/1.1/schema/qudt#>
PREFIX gleif-L1: <https://www.gleif.org/ontology/L1/>
PREFIX qudt-1-1: <http://qudt.org/1.1/schema/qudt#>

SELECT ?unit ?unitname ?cap ?share

WHERE
{
  ?unit a enbp:ProductionUnit; wikidata:P298 "CHE".
  OPTIONAL {?unit enbp:hasName ?unitname }
  OPTIONAL {?unit enbp:nameplateCapacity ?nameplate.  ?nameplate qudt-1-1:value ?cap}
  OPTIONAL {?owner a enbp:OwnershipInformation; enbp:ownershipOf ?unit; enbp:ownershipShare ?share}
}
```

Example: **List the sum of all capacities in a given country.** Run the following statement to calculate the sum of all capacities in a specific country. Here, Austria - AUS - is selected.

```
PREFIX schema: <http://schema.org/>
PREFIX dbo: <http://dbpedia.org/ontology/>
PREFIX enbp: <http://www.your-domain/>
PREFIX wikidata: <https://www.wikidata.org/entity/>
PREFIX qudt-1-1: <http://qudt.org/1.1/schema/qudt#>
PREFIX gleif-L1: <https://www.gleif.org/ontology/L1/>
PREFIX qudt-1-1: <http://qudt.org/1.1/schema/qudt#>

SELECT (SUM(COALESCE(?cap,0)) AS ?Total)
WHERE
{
  ?unit a enbp:ProductionUnit; wikidata:P298 "AUT"; enbp:nameplateCapacity ?nameplate.
  OPTIONAL {?unit enbp:hasName ?unitname}
  OPTIONAL {?nameplate qudt-1-1:value ?cap}
}
```

Example: **List of capacities according to SIEC type.** To list all capacities in a chosen country according to Standard Industrial Classification, run the following script. Here, Poland - POL - is selected.

```
PREFIX schema: <http://schema.org/>
PREFIX dbo: <http://dbpedia.org/ontology/>
PREFIX enbp: <http://www.your-domain/>
PREFIX wikidata: <https://www.wikidata.org/entity/>
PREFIX qudt-1-1: <http://qudt.org/1.1/schema/qudt#>
```

```
PREFIX gleif-L1: <https://www.gleif.org/ontology/L1/>
PREFIX qudt-1-1: <http://qudt.org/1.1/schema/qudt#>
PREFIX siec: <http://dd.eionet.europa.eu/vocabulary/eurostat/siec/>

SELECT ?unit ?unitname ?cap
WHERE
{
  ?unit a enbp:ProductionUnit; wikidata:P298 "POL"; enbp:hasSIEC siec:R5300.
  OPTIONAL {?unit enbp:hasName ?unitname }
  OPTIONAL {?unit enbp:nameplateCapacity ?nameplate.  ?nameplate qudt-1-1:value ?cap}
}
```

Example: **List membership information for a specific country.** Run the script below to get time-resolved membership counts for a given country. Here, Malta - MLT - is selected.

```
PREFIX schema: <http://schema.org/>
PREFIX dbo: <http://dbpedia.org/ontology/>
PREFIX enbp: <http://www.your-domain/>
PREFIX wikidata: <https://www.wikidata.org/entity/>

SELECT ?cai ?members ?date
WHERE
{
  ?cai a schema:Organization; wikidata:P298 "MLT" .
  OPTIONAL {?event a enbp:MembershipCount; wikidata:P2124 ?members; enbp:membersOf ?cai .}
  OPTIONAL {?event a enbp:MembershipCount; wikidata:P585 ?date; enbp:membersOf ?cai .}
}
```

Example: **List financial facts in a given country.** Here, Romania - ROU - is selected.

```
PREFIX schema: <http://schema.org/>
PREFIX dbo: <http://dbpedia.org/ontology/>
PREFIX enbp: <http://www.your-domain/>
PREFIX wikidata: <https://www.wikidata.org/entity/>
PREFIX xbrll: <https://w3id.org/vocab/xbrll#>

SELECT ?cai ?fact ?concept ?date ?value
WHERE
{
  ?cai a schema:Organization; wikidata:P298 "ROU" .
  ?fact a xbrll:Fact; xbrll:hasEntity ?cai.
  OPTIONAL {?fact wikidata:P585 ?date; }
  OPTIONAL {?fact xbrll:concept ?concept}
  OPTIONAL {?fact xbrll:value ?value}
}
```

Example: **List all economic activities for initiatives in a given country.** Run this query for owner and production unit vis-a-vis and adapt by changing the country code. Here, Austria - AUT - is selected.

```
PREFIX schema: <http://schema.org/>
PREFIX dbo: <http://dbpedia.org/ontology/>
PREFIX enbp: <http://www.your-domain/>
PREFIX wikidata: <https://www.wikidata.org/entity/>
PREFIX qudt-1-1: <http://qudt.org/1.1/schema/qudt#>
```

```
SELECT ?cai ?economic_activity ?eco_code ?eco_text ?NACE
WHERE
{
  ?cai wikidata:P298 "AUT"; enbp:hasEconomicActivity ?economic_activity.
  OPTIONAL {?economic_activity enbp:nationalIndustrialClassificationCode ?eco_code}
  OPTIONAL {?economic_activity enbp:nationalIndustrialClassificationText ?eco_text}
  OPTIONAL {?economic_activity enbp:hasNACE ?NACE}
}
```

Example: **List financial investments and share for initiatives and units in a given country.** Here, Belgium - BEL - is used.

```
PREFIX schema: <http://schema.org/>
PREFIX dbo: <http://dbpedia.org/ontology/>
PREFIX enbp: <http://www.your-domain/>
PREFIX wikidata: <https://www.wikidata.org/entity/>
PREFIX xbrll: <https://w3id.org/vocab/xbrll#>

SELECT ?buy ?cai ?cainame ?unit ?unitname ?date ?price ?currency
WHERE
{
  ?cai a schema:Organization; wikidata:P298 "BEL" .
  ?buy a schema:BuyAction; schema:agent ?cai; schema:object ?unit.
  OPTIONAL {?buy schema:endTime ?date}
  OPTIONAL {?buy schema:price ?price}
  OPTIONAL {?buy schema:priceCurrency ?currency}
  OPTIONAL {?cai schema:name ?cainame}
  OPTIONAL {?unit enbp:hasName ?unitname}
  OPTIONAL {?owner a enbp:OwnershipInformation; enbp:ownershipOf ?unit;   enbp:ownershipShare ?share}
}
```

Example: **Return status for initiatives in a given country.** Adapt by changing the country code; here, the Czech Republic - CZE - is used. Can be adapted in a similar fashion to return source, makes offer, and contributor information.

```
PREFIX schema: <http://schema.org/>
PREFIX dbo: <http://dbpedia.org/ontology/>
PREFIX enbp: <http://www.your-domain/>
PREFIX wikidata: <https://www.wikidata.org/entity/>
PREFIX sc: <http://purl.org/science/owl/sciencecommons/>
PREFIX geo: <http://www.w3.org/2003/01/geo/wgs84_pos#>
PREFIX dc: <http://purl.org/dc/elements/1.1/>

SELECT ?cainame ?status
{
 ?cai a schema:Organization; wikidata:P298 "CZE"; schema:name ?cainame; enbp:status ?status.
#  ?cai a schema:Organization; wikidata:P298 "CZE"; schema:name ?cainame; dc:source ?status.
#  ?cai a schema:Organization; wikidata:P298 "CZE"; schema:name ?cainame; dc:contributor ?status.
# ?cai a schema:Organization; wikidata:P298 "CZE"; schema:name ?cainame; schema:makesOffer ?status.
}
```

Useful SPARQL commands for database queries, initiative-level

<u>*Example:*</u> **List all properties and objects for a given initiative.** Run the script below inserting the desired initiative. Here, *cai_aut_1* is selected.

```
PREFIX schema: <http://schema.org/>
PREFIX dbo: <http://dbpedia.org/ontology/>
PREFIX enbp: <http://www.your-domain/>
PREFIX wikidata: <https://www.wikidata.org/entity/>

SELECT ?s ?p ?o

{
  enbp:cai_aut_1 ?p ?o
}
```

<u>*Example:*</u> **List all production units in a given country for a selected initiative.** Here, *cai_aut_93* is selected.

```
PREFIX schema: <http://schema.org/>
PREFIX dbo: <http://dbpedia.org/ontology/>
PREFIX enbp: <http://www.your-domain/>
PREFIX wikidata: <https://www.wikidata.org/entity/>

SELECT ?unit ?unitname

{
  ?unit a enbp:ProductionUnit; enbp:hasName ?unitname.
  ?ownership a enbp:OwnershipInformation; enbp:ownershipBy enbp:cai_aut_93; enbp:ownershipOf ?unit.
}
```

<u>*Example:*</u> **List the number and names of production units along with capacities for a specific initiative in a given country.** Run the script to get a list of all production unit types and capacities for a selected initiative. Here, *cai_deu_68* is selected.

```
PREFIX schema: <http://schema.org/>
PREFIX dbo: <http://dbpedia.org/ontology/>
PREFIX enbp: <http://www.your-domain/>
PREFIX wikidata: <https://www.wikidata.org/entity/>
PREFIX qudt-1-1: <http://qudt.org/1.1/schema/qudt#>
PREFIX gleif-L1: <https://www.gleif.org/ontology/L1/>
PREFIX qudt-1-1: <http://qudt.org/1.1/schema/qudt#>

SELECT ?unit ?unitname ?cainame ?cap

{
  ?unit a enbp:ProductionUnit; enbp:hasName ?unitname.
  ?ownership a enbp:OwnershipInformation; enbp:ownershipBy  enbp:cai_deu_68; enbp:ownershipOf ?unit.
  enbp:cai_deu_68 schema:name ?cainame.
   OPTIONAL {?unit enbp:nameplateCapacity ?nameplate.  ?nameplate qudt-1-1:value ?cap}
}
```

Other useful SPARQL commands for database queries

Example: **Selecting information that exactly matches a string (e.g., part of a street name)**. To select information that contains a particular string or substring, the FILTER keyword can be used. Building on the example before, let us assume that you query for a particular street. In particular, we want to select all the information about name, street, etc. with street names containing the string "Slovenska". To do so, the previous statement is extended by the line FILTER(regex(?street, "Sloveska","i")). The entire query becomes:

```
PREFIX dc: <http://purl.org/dc/elements/1.1/>
PREFIX enbp:  <http://www.your-domain/>
PREFIX wikidata: <https://www.wikidata.org/entity/>
PREFIX schema: <http://schema.org/>
PREFIX gleif-L1: <https://www.gleif.org/ontology/L1/>

SELECT ?cainame ?street ?city ?postal ?status ?gleif

WHERE {
        ?cai a enbp: CAI.
        ?cai wikidata:P298 "SVN".
        ?cai schema:name ?cainame.
        ?cai schema:streetAddress ?street.
        ?cai schema:addressLocality ?city.
        ?cai schema:postalCode ?postal.
        ?cai enbp: status ?status.
  FILTER (regex(?street,"Slovenska","i"))
  OPTIONAL { ?cai gleif-L1:hasLegalForm ?gleif}
}
```

The query above returns only entries where values for all objects are given. The variant below would list entries with missing address information:

```
SELECT ?cainame ?street ?city ?postal ?status ?gleif

WHERE {
        ?cai a enbp: CAI.
        ?cai wikidata:P298 "SVN".
        ?cai schema:name ?cainame.
          OPTIONAL { ?cai schema:streetAddress ?street }
          OPTIONAL { ?cai schema:addressLocality ?city }
          OPTIONAL { ?cai schema:postalCode ?postal }
          ?cai enbp: status ?status.
          OPTIONAL { ?cai gleif-L1:hasLegalForm ?gleif}
}
```

Instead of the 8 results returned before, now only 3 results are returned where the street address contains the string "Slovenska". In fact, all three initiatives are located at the same street address, Slovenska cesta 24.

Example: **Search for production information.** To query information on all PV plants, use the following statement:

```
PREFIX dc: <http://purl.org/dc/elements/1.1/>
```

```
PREFIX enbp:  <http://www.your-domain/>
PREFIX wikidata: <https://www.wikidata.org/entity/>
PREFIX schema: <http://schema.org/>
PREFIX gleif-L1: <https://www.gleif.org/ontology/L1/>

SELECT DISTINCT ?s

WHERE {
        ?s a enbp: PVPlant; ?p ?o
}
```

If information is required for all production units, use "ProductionUnit" instead of "PVPlant". The query can also be specialized if capacity information is searched after. The capacity information is not reported with the upper statement because it is encapsulated in a blank node. Please use the following statement for such a purpose:

```
PREFIX dc: <http://purl.org/dc/elements/1.1/>
PREFIX enbp:  <http://www.your-domain/>
PREFIX wikidata: <https://www.wikidata.org/entity/>
PREFIX schema: <http://schema.org/>
PREFIX gleif-L1: <https://www.gleif.org/ontology/L1/>
PREFIX qudt-1-1: <http://qudt.org/1.1/schema/qudt#>
PREFIX qudt-unit-1-1: <http://qudt.org/1.1/vocab/unit#>

SELECT DISTINCT ?s ?name ?date ?cap

WHERE {
        ?s a enbp: ProductionUnit; wikidata:P298 "HRV"; enbp: hasName ?name.
  OPTIONAL {?s enbp: commissionDate ?date}
  OPTIONAL {?s enbp: nameplateCapacity ?nameplate. ?nameplate qudt-1-1:value ?cap.}
}
```

The OPTIONAL keyword is used to make sure that results are reported even if no date or capacity is set.

Extended PREFIX and variable lists for applied standards

List of helpful prefixes

PREFIX co: <http://purl.org/ontology/co/core#>
PREFIX com: <http://purl.org/commerce#>
PREFIX rdf: <http://www.w3.org/1999/02/22-rdf-syntax-ns#>
PREFIX ma: <http://www.w3.org/ns/ma-ont#>
PREFIX dc: <http://purl.org/dc/elements/1.1/>
PREFIX schema: <http://schema.org/>
PREFIX enbp: <http://www.your-domain/>
PREFIX dbo: <http://dbpedia.org/ontology/>
PREFIX dbr: <http://dbpedia.org/resource/>
PREFIX geo: <http://www.w3.org/2003/01/geo/wgs84_pos#>
PREFIX gleif: <https://www.gleif.org/ontology/Base/>
PREFIX gleif-elf: <https://www.gleif.org/ontology/EntityLegalForm-v1.0/EntityLegalForm/>

PREFIX gleif-L1: <https://www.gleif.org/ontology/L1/>
PREFIX siec: <http://dd.eionet.europa.eu/vocabulary/eurostat/siec/>
PREFIX nace: <https://dd.eionet.europa.eu/vocabulary/eurostat/nace_r2/>
PREFIX country: <http://dd.eionet.europa.eu/vocabulary/common/countries/>
PREFIX qudt-1-1: <http://qudt.org/1.1/schema/qudt#>
PREFIX qudt-unit-1-1: <http://qudt.org/1.1/vocab/unit#>
PREFIX orcid: <http://www.orcid.org/>
PREFIX wikidata: <https://www.wikidata.org/entity/>
PREFIX grel: <http://users.ugent.be/~bjdmeest/function/grel.ttl#>
PREFIX xbrll: <https://w3id.org/vocab/xbrll#>
PREFIX geop: <http://aims.fao.org/aos/geopolitical.owl#>
PREFIX rov: <http://www.w3.org/ns/regorg#>
PREFIX xsd: <http://www.w3.org/2001/XMLSchema#>
PREFIX oeo: <https://openenergy-platform.org/ontology/oeo/>

List of helpful variables for administrative information

**Variables**	**Predicates**
Name	schema:name
National identifier	schema:identifier
Status	enbp: status
Country	wikidata:P298 “xxx”; schema:addressCountry “xx”; wikidata:P17 wikidata:Qxxx
Street address	schema:streetAddress
City	schema:addressLocality
Postal code	schema:postalCode
Latitude	geo:lat
Longitude	geo:long
Website	schema:url
Contact	schema:contactPoint
Founding date	schema:foundingDate
Dissolution date	schema:dissolutionDate
Legal form, text	wikidata:P1454
Legal form, GLEIF	gleif-L1:hasLegalForm

Economic activity, national code	enbp:nationalIndustrialClassificationCode
Economic activity, text	enbp:nationalIndustrialClassificationText
NACE code	enbp:hasNACE, wikidata:P4496
Activities	schema:makesOffer
Energy as primary purpose	enbp:energyIsPrimaryActivity
Purpose statement	enbp:hasPurpose
Comments	enbp:comments
Creator of ttl file	dc:creator
Contributor to data	dc:contributor
Publisher	dc:publisher
Sources	dc:source
Date	dc:date

List of helpful variables for production information

Variable	Predicate
name of installation	enbp:hasName
status	enbp:status
country	wikidata:P298
technology	enbp:technology
SIEC	enbp:hasSIEC
latitude	geo:lat
longitude	geo:long
Street address	schema:streetAddress
Municipality	schema:addressLocality
Postal code	schema:postalCode
Commissioning date	wikidata:P729

Decommissioning date	wikidata:P730
Nameplate Capacity	wikidata:P2019
Estimated yearly generation	enbp:estimatedYearlyGeneration
Device specification	enbp:deviceSpecification
Comment	enbp:comment
Creator	dc:creator
Contributor	dc:contributor
Publisher	dc:publisher
Source	dc:source
Date	dc:date

List of helpful variables for events and event classes

Variable	Predicate	Event class
Membership count, value	wikidata:P2124	enbp:MembershipCount
Membership count, date	wikidata:P585	enbp:MembershipCount
Membership count, initiative	enbp:membersOf	enbp:MembershipCount
Number of Employees, value	wikidata:P1128	enbp:EmployeeCount
Number of Employees, date	wikidata:P585	enbp:EmployeeCount
Number of Employees, initiative	enbp:employeesOf	enbp:EmployeeCount
Number of Customers, value	enbp:numberOfCustomers	enbp:CustomerCount
Number of Customers, date	wikidata:P585	enbp:CustomerCount
Number of Customers, initiative	enbp:customersOf	enbp:CustomerCount
Annual generation	wikidata:P4131	enbp:ElectricityGenerated

List of national identifier codes taken from wikidata

Country	Id name	Wikidata ID
Denmark	CVR number	P1059
Netherlands	KvK number	P3220

Austria	Firmenbuchnummer	P5285
Czech	Czech Registration ID	P4156
Belgium	Belgium enterprise number	P3376
Estonia	Business Registry code	P6518
Latvia	Unified registration number	P8053
Slovakia	Registration ID	P8174
Norway	Organization number	P2333
France	SIREN number	P1616
Great Britain	Companies House company ID	P2622
Ireland	Companies Registration Office (Ireland) Registration Number	P9679
Switzerland	Swiss Enterprise Identification Number	P4829
Sweden	Swedish Organization Number	P6460
